# Structure‐Based Varieties of Polymeric Nanocarriers and Influences of Their Physicochemical Properties on Drug Delivery Profiles

**DOI:** 10.1002/advs.202105373

**Published:** 2022-02-03

**Authors:** Ranjit De, Manoj Kumar Mahata, Kyong‐Tai Kim

**Affiliations:** ^1^ Laboratory of Molecular Neurophysiology Department of Life Sciences Pohang University of Science and Technology (POSTECH) 77 Cheongam‐Ro Pohang Gyeongbuk 37673 South Korea; ^2^ Division of Integrative Biosciences and Biotechnology (IBB) Pohang University of Science and Technology (POSTECH) 77 Cheongam‐Ro Pohang Gyeongbuk 37673 South Korea; ^3^ Drittes Physikalisches Institut ‐ Biophysik Georg‐August‐Universität Göttingen Friedrich‐Hund‐Platz 1 Göttingen 37077 Germany

**Keywords:** multi‐drug delivery, multi‐stimuli‐responsive, nanomaterials, polymers, self‐assembly

## Abstract

Carriers are equally important as drugs. They can substantially improve bioavailability of cargos and safeguard healthy cells from toxic effects of certain therapeutics. Recently, polymeric nanocarriers (PNCs) have achieved significant success in delivering drugs not only to cells but also to subcellular organelles. Variety of natural sources, availability of different synthetic routes, versatile molecular architectures, exploitable physicochemical properties, biocompatibility, and biodegradability have presented polymers as one of the most desired materials for nanocarrier design. Recent innovative concepts and advances in PNC‐associated nanotechnology are providing unprecedented opportunities to engineer nanocarriers and their functions. The efficiency of therapeutic loading has got considerably increased. Structural design‐based varieties of PNCs are widely employed for the delivery of small therapeutic molecules to genes, and proteins. PNCs have gained ever‐increasing attention and certainly paves the way to develop advanced nanomedicines. This article presents a comprehensive investigation of structural design‐based varieties of PNCs and the influences of their physicochemical properties on drug delivery profiles with perspectives highlighting the inevitability of incorporating both the multi‐stimuli‐responsive and multi‐drug delivery properties in a single carrier to design intelligent PNCs as new and emerging research directions in this rapidly developing area.

## Graphical abstract

Ease of designing polymeric nanocarriers (PNCs) in various structures is one of the prominent reasons for their widespread use in biomedical applications. This review focuses on exploring structure‐based varieties of PNCs and influences of their physicochemical properties on their drug delivery profiles. It also highlights the use of multi‐stimuli responsive multi‐drug delivery PNCs as new and emerging research direction.

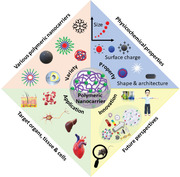

## 1 Introduction

**Figure 1:**
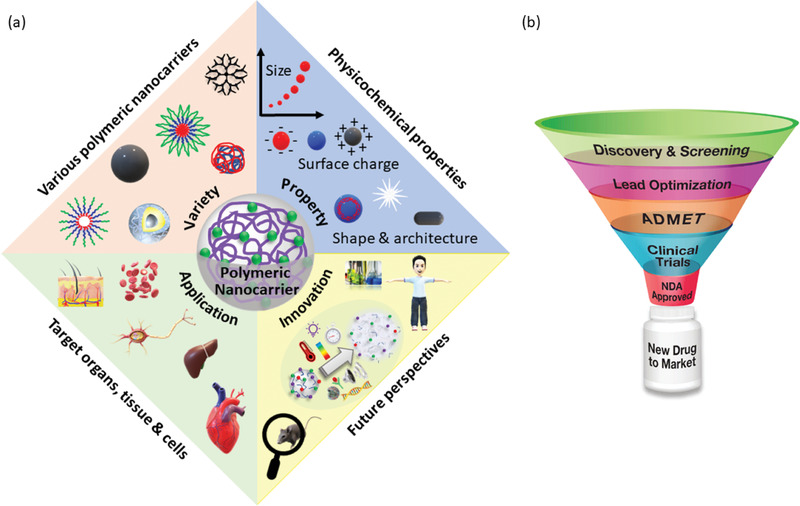
a) A schematic presentation overviewing variety, property, applications, and future perspectives of polymeric nanocarriers discussed in this study. b) Multiple steps involved in bringing a new drug into clinical practice. Abbreviations. ADMET: Absorption, Distribution, Metabolism, Excretion, and Toxicity. NDA: New Drug Application. (Reproduced with permission from Enzo Life Sciences. Innovative Tools for Accelerating Drug Discovery. Retrieved from https://www.enzolifesciences.com/browse/drug‐discovery/).

Developments in nanotechnology have contributed to substantial progress in designing precision nanocarriers for advanced drug delivery.^[^ ^1^, ^2^ ^]^ Subsequently, these nanocarriers have shown tremendous potential to overcome the limitations of otherwise solo drugs and navigate through biological barriers to achieve targeted delivery even to the organelles located at the subcellular region.^[^ ^3^, ^4^ ^]^ Designing personalized medications loaded with precision therapeutics, to increase efficacy of targeted delivery, is a key goal of advanced nanomedicine. This review presents a comprehensive investigation of structural design‐based varieties of polymeric nanocarriers (PNCs) and the influences of their physicochemical properties on drug delivery profiles with the perspectives highlighting multi‐stimuli responsive multi‐drug delivery nanocarriers as new and emerging research directions in this area. A succinct presentation of the findings about influences of these physicochemical properties on the success of achieving targeted delivery along with their future perspectives are illustrated in **Figure** **1**a.

### 1.1 Significance of Drug Carriers

Development of a new drug requires substantial amount of research and investment before it can be approved for clinical use. It has to go through various steps as presented in Figure 1b. Only about 1 in 10 000 drug candidates gets selected for marketing while the rest are discarded. Thus, introduction of one drug molecule to clinical practice can cost as much as $100 million.^[^ ^5^, ^6^, ^7^, ^8^ ^]^ Here, induction of drug delivery carriers plays pivotal role to significantly increase the number of potential candidate drugs by protecting those against harsh conditions, extending lifetime in the circulatory system, facilitating targeted delivery, and controlled release, which subsequently improve their pharmacokinetics and pharmacodynamics.^[^ ^9^, ^10^, ^11^, ^12^ ^]^ Thus, in biomedicine design and delivery science, drug carrier is as important as the drug itself.^[^ ^13^ ^]^ Nevertheless, these carriers are to withstand various circumstances, such as, changes in environmental pH and ionic concentrations, enzymatic actions, heat, and influences of crowding agents. For example, drugs administered orally face gastrointestinal hurdles posed by enzymatic attack, variable pH, etc.^[^ ^14^ ^]^ Delivery to central nervous system faces challenges posed by blood‐brain barrier (BBB).^[^ ^15^, ^16^, ^17^, ^18^ ^]^ A recent study showed, only about 1% of nanocarriers can accumulate with high permeability and retention in xenografted tumors; this low rate is possibly due to multiple physiological barriers.^[^ ^19^, ^20^ ^]^ These challenges indicate that the nanocarriers must be substantially improved.

Progress in modern nanotechnologies along with continuous efforts of improving drug delivery carriers are transforming these challenges into opportunities.^[^ ^21^, ^22^, ^23^ ^]^ However, it has always been a formidable task to develop case specific carriers as one must consider both the drug properties and delivery process which vary from disease to disease and their complexity. Consequently, carriers of various shapes, sizes, materials, and properties must be developed constantly.^[^ ^1^ ^]^ Among various conventional ones, carriers of nano dimensions are noteworthy as they can exhibit efficient penetration through cell membrane, easy passage in the circulatory system, high loading efficacy, and promising capability to permeate through physiological barriers (skin, BBB), which are often unmet by the traditional macro‐sized carriers.^[^ ^16^, ^24^, ^25^, ^26^ ^]^ Furthermore, multifunctional nanocarriers are also emerging as remarkable canditates which can perform both the works of diagnosis and drug delivery. For example, a multifunctional drug delivery carrier designed by Nasongkla et al. has demonstrated its use for disease diagnosis via magnetic resonance imaging and treatment through drug delivery.^[^ ^27^ ^]^ Recently, as the research progresses, nanocarriers are being designed to co‐load multiple drugs at a time and simultaneously be able to respond to multiple stimuli for the application of site‐specific drug release.^[^ ^28^, ^29^, ^30^ ^]^ There are excellent review articles covering various nanocarriers, their design strategies, and applications.^[^ ^31^, ^32^, ^33^, ^34^ ^]^ Hence, this study is confined to structure‐based varieties of PNCs and the role of their physicochemical properties that are exploited to improve drug delivery profile.

### 1.2 Nanotechnology for Nanocarrier Design

Nanobiotechnology has become an integral part of daily life and essential in sectors like healthcare, beauty products, and food industry.^[^ ^35^, ^36^, ^37^, ^38^ ^]^ According to European Medicines Agency, nanotechnology serves as interface in translating fundamental findings of biology to their applications in healthcare.^[^ ^39^ ^]^ Nanocarriers can overcome various limitations, which are otherwise faced by conventional delivery carriers, and accomplish superior biodistribution, efficient intra‐cellular trafficking, and organelle specific targeting.^[^ ^1^ ^]^ These nanocarriers have also exhibited potential contribution to achieve high success in disease diagnosis and treatment specificity.

Motivated by the irrefutable prospective of nanotechnology in biomedicine, National Nanotechnology Initiative (NNI) was set up in 2000 by the US National Science and Technology Council (NSTC) to lead the revolution in healthcare industry.^[^ ^40^ ^]^ Efforts by various such institutions have encouraged expanded research activities, which have yielded numerous developments in nanocarrier design, as evident through the increased numbers of publications and patents. Nevertheless, the number of nanomedicines available for clinical practice is much less than the actual requirement. This gap is due to the challenges of transferring knowledge obtained by studying animal models to their implementation in human studies.^[^ ^41^, ^42^, ^43^ ^]^ Heterogeneity among patients is another constrain that extends this problem. Although nanomaterials can provide versatile benefit, engineering their designs and synthesizing them to meet each need is a daunting task, however, it is an utmost necessity.

A suitable nanocarrier can reduce side effects of certain therapeutics by keeping away from untargeted organs and tissues.^[^ ^44^ ^]^ Additionally, due to high surface‐area‐to‐volume ratio, nanocarriers can show high loading efficacy. These nanostructures have excellent capability to alter several physical properties of drugs, such as, their solubility, diffusion, bioavailability, immune response, and release profile, which can be regulated by modifying their shape, size, composition, and surface property.^[^ ^45^, ^46^ ^]^ Simultaneously, these carriers can impart protection against premature degradation of cargos having delicate structures, hence they are also instrumental in designing protein‐based therapeutics, which are often delicate.^[^ ^47^, ^48^ ^]^ Thus, the progress of nanotechnology in drug delivery carrier design has offered the potential to revolutionize pharmaceutical industry and provide answers to several unresolved questions.^[^ ^49^ ^]^

### 1.3 Advantages of Nanocarriers in Biomedicine

Nanocarriers can be defined as the particles having at least one of their dimensions within 1–100 nm range and engaged in transporting another substance, such as, a drug, gene, protein, etc.^[^ ^50^ ^]^ Commonly utilized carriers include micelles, emulsions, carbon‐based substances, liposomes, gels, and composites.^[^ ^51^ ^]^ Some literatures loosely define nanocarriers as the particles that have sub‐micrometer dimension (100–1000 nm).^[^ ^52^, ^53^ ^]^ However, in the context of drug delivery and due to the tiny diameter of microcapillaries, the particles of dimension <200 nm are considered as nanocarriers.^[^ ^54^ ^]^ Moreover, the efficiency of uptake by cells via endocytosis is best when the size of therapeutic loaded carriers are within this range.^[^ ^25^, ^55^, ^56^, ^57^, ^58^, ^59^ ^]^ Facilitated by their nano dimension, these carriers can convey therapeutics to almost any destinations in the body. These have also been found instrumental in facilitating drug permeation across BBB in central nervous system.^[^ ^60^, ^61^ ^]^ With the assistance of nanocarriers, macromolecules such as, proteins, can also get successfully transported to cytosol and thereby overcomes the problem of getting aggregated at the cell membrane. In some cases, nanocarriers can also function as nano‐templates to control the overall size of drug‐loaded carriers. Furthermore, nanoparticles with inherent fluorescent properties are contributing to disease diagnosis and monitoring via bioimaging.^[^ ^62^ ^]^

### 1.4 Section Summary

Use of carriers can improve pharmacokinetics and pharmacodynamics of drugs, and thereby widening the choice of drugs for inclusion in clinical applications. Carriers of nanoscale dimensions further improve the potential of drug molecules through easy passage in the circulatory system and mucous layers with improved targeted delivery. Furthermore, progress in nanotechnology has facilitated fabricating of multifunctional nanocarriers. Thus, nanotechnology‐based improvements in biomedicine have permitted researchers to extend successful applications of nano formulations in various clinical complications with the aim of obtaining highly effective remedies. Advances in nanotechnology have provided various techniques to control architecture and dispersity of numerous small‐ and macro‐molecules of therapeutic prominence and have thereby integrated multiple functions of nanomaterials in nanomedicine.

## 2 Significance of Polymers in Nanocarrier Design

**Figure 2:**
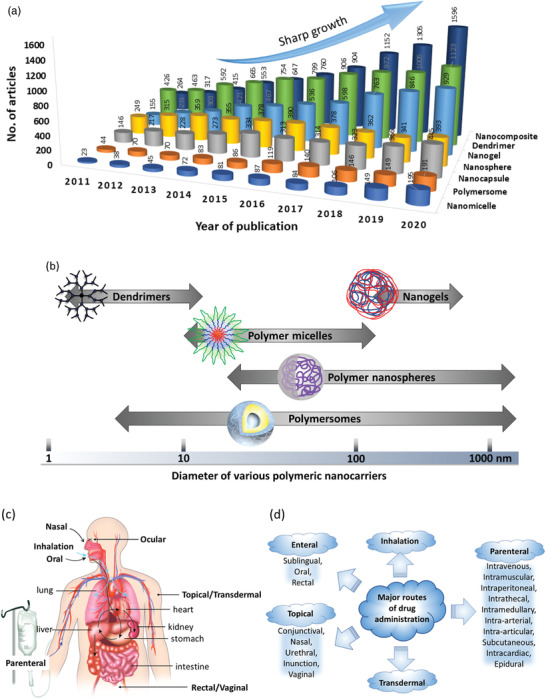
a) Number of articles published per year on various polymeric carriers (Database: Scopus. Keywords used for the searches: “drug delivery system” + “polymer” and then each of the following nanocarriers, “nanocomposite;” “dendrimer;” “nanogel;” “nanosphere;” “nanocapsule;” “polymersome;” “nanomicelle”). b) Size ranges of various polymeric nanocarriers (PNCs). c) Various anatomic routes for drug administration. Reproduced with permission.^[^ ^103^ ^]^ Copyright 2018, Elsevier. Reproduced with permission from National institute of general medical sciences image., A drug's life in the body (without labels), https://images.nigms.nih.gov/Pages/DetailPage.aspx?imageID2=2527, accessed date: 9 January 2022. d) Examples of major routes for drug administration.

Polymers have gained enormous attention for designing nanocarriers and their prospects in medical applications are vast.^[^ ^63^, ^64^ ^]^ They are macromolecules composed of multiple repeating subunits and have the advantage of being able to host wide range of functional groups.^[^ ^65^ ^]^ This phenomenon presents them to be explored for diverse bioapplications. Although polymers are macro molecules, they can be used to construct therapeutic delivery carriers of nano dimensions. Moreover, biodegradable property in many of such polymers makes them the most promising candidate material as they can be used without the concern of clearance from the body.^[^ ^66^ ^]^ Hence, use of biodegradable polymers to prepare nanocarriers is one of the most desired schemes.^[^ ^67^, ^68^, ^69^ ^]^ Lifetime enhancement and superior targeted delivery of drugs encapsulated in PNCs are commendable. The molecular architectures of polymers can also play significant role as they can be exploited to engineer nanoparticles that have various morphologies and architectures.^[^ ^70^, ^71^ ^]^ With the progress in nanotechnology, new possibilities of designing such nanocarriers have attracted interests of many researchers. Stimuli‐responsive polymers are potential candidates to accomplish controlled release of therapeutics at targeted sites.^[^ ^72^, ^73^ ^]^ Converting these macromolecules to nanocarriers can add remarkable potential to drug molecules. Polymers can tune the physicochemical properties and add multifunctionality to carriers.^[^ ^74^ ^]^ As mentioned earlier, due to the tiny dimensions of PNCs, they can pass through very small capillaries and mucous layers into the targeted cells, as well as, the intracellular organelles. Nanosized polymeric structures such as micelles, vesicles, gels, capsules, dendrimers, composites, etc., have enormously pliable properties, and are therefore being increasingly investigated as vehicles to deliver therapeutics. The increase in investigations of various types of PNCs for drug delivery is noticeable through the growing number of literatures as presented in **Figure** **2**a. During the recent five years, this growth rate has further increased.

Polymers can blend with other high‐ or low‐molecular weight compounds and can be tailored to form composites for desired applications. Unlike many other materials, it has been decades that these macromolecules are being engaged in medicinal chemistry. They are also widely used as stabilizers, taste‐making factors, and protective agents in oral drug formulations. They can bind with particles of solid dosage form and change their flow characteristics to liquid dosage form. Similarly, polymer‐to particle‐transition also influences their flow properties exploitable for drug delivery purposes.^[^ ^75^ ^]^ Their promising surface and bulk properties have exceptional contribution in drug design and delivery. Colloidal particles of these materials show efficient drug loading, expanded lifetime, augmented shelf‐life, and sustained release.^[^ ^76^ ^]^ Thus, polymer‐based nanostructures play crucial role in formulating advanced nanomedicines.

Polymers have also shown advantages over various other biofriendly materials, such as, DNA, proteins, etc. Although the biocompatibility and biodegradability of DNA and protein‐like materials present them as promising candidates for nanocarrier design, engineering these materials to make nanocarriers is highly challenging. DNA‐based nanostructure development requires rational designing which is often difficult as their modification via sequence‐specific interactions demands high level of expertise.^[^ ^77^ ^]^ Furthermore, improving the stability of such nanostructures requires additional attention.^[^ ^78^ ^]^ Even though DNA molecules are naturally biocompatible, they may produce acute inflammatory response.^[^ ^79^ ^]^ To increase drug loading efficacy, the dynamic structure of DNA often requires modification. Characterizations of DNA nanostructures have also provided conflicting results due to the lack of standardized techniques.^[^ ^80^ ^]^ The variety of drugs that can be loaded onto DNA nanostructures is limited. Additionally, it is challenging to make them stable at the ambient temperature. DNAs are often functionalized by proteins to improve their drug delivery properties. Although such functionalization can enhance loading efficacy of specific immunotherapeutic and vaccine adjuvants, improving their stability remains a daunting task.^[^ ^80^ ^]^ It has been proposed that conjugating polymers with DNA can produce various nanostructures and can improve their stability.^[^ ^81^ ^]^ Furthermore, the polymer molecules are comparatively stable, and their morphology can introduce both flexibility, as well as, rigidity without resulting any undesired change in their physicochemical properties. Even though DNA‐based materials are biodegradable, comprehensive assessments are essential prior to their clinical trial as it may raise the concern of biosafety. Like DNA‐based materials, solo proteins are also being studied to design drug carriers. However, they also suffer from stability issues as elevated temperature can denature them. Moreover, their circulation half‐life is often short as they are susceptible to degradation caused by enzymes present in the circulatory system. This often leads to failure in achieving targeted delivery. Large scale production and sterilization of these biodegradable materials are often difficult. To address these challenges, polymers are being investigated to wrap such sensitive materials or conjugate with them to prohibit their easy denaturation and thereby improve stability.^[^ ^82^, ^83^ ^]^ Various types of possible interactions between the functional groups of polymers and protein molecules are being explored to play the crucial role in resolving this. Thus, as the polymer molecules themselves have huge potential in designing PNCs for drug delivery, they can also contribute to protect and stabilize DNA, protein‐based nanocarriers.^[^ ^84^ ^]^

Acceptance of PNCs for clinical applications are in the rise.^[^ ^63^ ^]^ Biodegradable PNCs are used to deliver drugs, formulate vaccines, stabilize drug emulsions, and serve as contrast agent during imaging.^[^ ^85^ ^]^ There are various FDA (Food and Drug Administration) approved polymers being used in clinical practices. The first FDA‐approved nano‐drug Doxil, which is the doxorubicin encapsulated nano liposome, has the coating of poly(ethylene glycol) (PEG) to enhance drug circulation half‐life required in cancer (Kaposi's sarcoma, recurrent ovarian cancer, etc.) treatment.^[^ ^86^ ^]^ The hormone leuprolide encapsulated in poly(lactic‐*co*‐glycolic) acid (PLGA) particles are used to treat endometriosis, as well as, advanced prostate cancer.^[^ ^87^ ^]^ Polymer‐based nanoparticles and nanostructures are also being used for cardiovascular disease treatment.^[^ ^88^ ^]^ Drug loaded PNCs have been employed to coat stents used for on‐site drug elution.^[^ ^89^ ^]^ PEG coated liposomal irinotecan (MM‐398/Onivyde) has been approved by FDA to treat various cancers in malignancies, breast, pancreatic, sarcomas, brain, etc.^[^ ^90^ ^]^ Iron loaded colloidal nanoparticles of dextran (DexFerrum) are used to treat iron deficient anemia, while iron loaded sucrose (Venofer) nanoparticles are used to treat anemia that may arise following the autologous stem cell transplantation.^[^ ^90^ ^]^ Polyglucose sorbitol carboxymethylether nanoparticles loaded with iron (Ferumoxytol) can be used to treat iron deficient anemia, as well as, for the imaging of brain metastases, lymph node metastases, neuroinflammation in epilepsy, multiple sclerosis, myocardial infarction, head and neck cancer, etc.^[^ ^90^ ^]^ Use of PNCs for imaging and theranostic purposes are also in the rise. To treat various other diseases, drug loaded PNCs have been approved to administer via different delivery routes, such as, dermal, transdermal, oral, mucosal, etc., depending upon the site of action.^[^ ^91^ ^]^ Thus, polymeric materials have huge potential for clinical applications.

Furthermore, recently the searches for multifunctional PNCs are in the rise. A reason behind this is the focus shift of present‐day medical science toward the use of multipurpose therapeutics.^[^ ^92^ ^]^ To address this demand, one of the solutions being emerged is the development of multi‐stimuli responsive multi‐drug delivery PNCs. Such multifunctional PNCs can respond to multiple stimuli and load/release multiple drugs. For example, there are PNCs being developed which can load multiple drugs and can respond to multiple stimuli, say pH and ultrasound, or temperature and pH, etc. At present development of such multifunctional PNCs are at the premature stage and needs further studies. A full translation of their performances from cellular level to the clinical application in patients has a long way to go. Here, it is to note that designing each multi‐functional PNC system is a crucial step toward the development of efficient personalized nanomedicine.

### 2.1 Structure‐Based Variety of Polymeric Nanocarriers

Researchers’ interests in the study of PNCs emerged mainly due to the advantages of personalized drug administration, increased bioavailability, sustained release from a single dose, and capability of safe carriage until delivered to the targeted site.^[^ ^93^, ^94^ ^]^ Traditional medication has numerous challenges like continuous administration of the medicine with a shorter half‐life, diminished patient consistency, high and ordinary peak‐valley plasma concentration‐time profile, and so on. As a result, in most of the cases targeted release remains unachieved. Because of these shortfalls, there is a rise in the altered and targeted medication practices. The traditional method of medication is gradually being replaced by the modern sophisticated approaches. Here, polymer nanostructures are playing decisive role in developing a modified and targeted medication conveyance system.^[^ ^95^ ^]^ Polymer nanostructures are solid colloidal materials composed of polymeric assembly and are ideally obtained from natural, synthetic or semi‐synthetic sources.^[^ ^96^ ^]^ They can load drugs, proteins, DNA, or RNA and defend them from denaturation and carry to targeted cells or tissue. To the matrix of polymer nanoparticles, the drug or any other compounds can get dissolved, attached, entrapped, or encapsulated, relying on different loading techniques.^[^ ^97^, ^98^ ^]^ Depending on the molecular architectures and methods of fabrication, these nanocarriers can be designed in various sizes (10–1000 nm), shapes (spherical, rod, cylindrical, star‐like), and structures (sphere, core‐shell, capsule, micelle, network) (Figure 2b). Such varieties of structure and associated physicochemical properties present these nanocarriers as promising candidates for a wide range of bioapplications, from disease diagnosis to cure.^[^ ^99^ ^]^

### 2.2 Significance of Polymeric Nanocarriers

PNCs are being widely used in many fields including anticancer drug design, mobilizing immune response against cancer or pathogens (tumor targeted immunotherapy, components for modern vaccines), regenerative medicine and topical applications, and treatment of lifestyle diseases.^[^ ^92^ ^]^ During the past decade, these PNCs have gained huge attention and become a prominent area of research. These carriers can enhance solubility of drugs and at the same time their polymeric materials can exhibit biocompatibility as well as biodegradability. They can be designed to incorporate both the hydrophilic and hydrophobic drugs. These PNCs can provide enhanced stability to the drugs, especially to the ones which are volatile and susceptible to heat or enzymes. Within PNCs, drugs can stay conjugated to or wrapped by polymer chains or get encapsulated within the nanostructures which lower the possibility of their nonspecific interactions with untargeted cells and tissues. These phenomena are advantageous to achieve targeted delivery. PNCs can also improve nonimmunogenicity. The ease of fabricating PNCs in aqueous media or mixed solvents can minimize impacts of various harsh condition parameters (e.g., solvent toxicity, high temperature, etc.) which otherwise had the risk of degrading molecular conformation and functions of loaded drugs. The large surface‐area‐to‐volume ratio, that is, volume specific surface area of PNCs allows drug molecules to access higher number of polymer functional groups available at the nanoparticle surface which is advantageous for enhanced drug‐polymer interactions and subsequently better loading. Furthermore, due to their nanoscale dimensions, PNCs can get easy passage through small capillaries and mucous layers resulting superior targeted delivery.^[^ ^100^ ^]^ PNCs composed of synthetic ABC‐type triblock copolymer, such as poly(ethylene glycol)‐*b*‐poly(2,4,6‐trimethoxybenzylidine‐1,1,1‐tris(hydromethylethane methacrylate)‐*b*‐poly(acrylic acid) is found to show high efficiency in loading and pH‐dependent intracellular release of doxorubicin hydrochloride.^[^ ^101^ ^]^ To manage and deliver drugs to a targeted site, the route adopted for the delivery is also an important factor for the therapeutics to achieve their full efficacy in disease treatment. Results can differ based on the routes opted as the efficacy of the drug also depends on the environment it encounters during its passage. Normally the administration is systemic but depending on the seriousness of disease or toxicity of drug, it may be administered directly to the affected location or sometimes they need to go through a specific route. A schematic presentation of various anatomic routes of administrating drugs, including the PNC‐based formulations, are presented in Figure 2c,d.^[^ ^102^, ^103^, ^104^ ^]^ Hydrophilic PNCs of size <4 nm can get rapidly excreted by kidneys, whereas those of sizes up to ≈200 nm can achieve longer circulation half‐life. In contrast, hydrophobic nanoparticles of size >200 nm can be taken up by reticuloendothelial system (RES). Hence, for a specific route of administration PNCs need to be designed exclusively keeping the challenges under consideration that it may face on its way to the delivery site.

Tacrolimus (TAC), an anti‐rejection drug, is generally used to avoid immunorejection of heart, kidney, or liver transplants and also as importance in ophthalmology. However, due to its poor penetration different nanocarriers have been tested. Laptova et al. have demonstrated that the use of polymeric nanomicelles as PNCs made of biodegradable and biocompatible methoxy‐poly(ethylene glycol)‐dihexyl substituted polylactide diblock copolymer can achieve targeted delivery of TAC to the epidermis and upper dermis.^[^ ^105^ ^]^ In another study, they has demonstrated the use of micellar PNCs comprised of diblock methoxy‐poly(ethylene glycol)‐poly(hexylsubstituted lactic acid) copolymer for the improved delivery of retinoic acid to treat acne.^[^ ^106^ ^]^ Castro et al. prepared positively charged PNCs composed of the polymer Eudragit RL100 to improve the ocular penetration of TAC during topical use.^[^ ^107^ ^]^ TAC‐PNCs with their mean size 104 ± 1 nm, positive surface charge and mucoadhesive characteristics induced appropriate properties for ocular use. Ryu et al. have developed a fast‐dissolving dry tablet to improve ocular drug availability, consisting of dexamethasone encapsulated within PLGA nanoparticles.^[^ ^108^ ^]^ It is found that alginate system consisting of PLGA nanocarriers increase the ocular drug bioavailability by 2.6 times compared to maxidexR. Yu et al. had successfully loaded an immunosuppressive agent, cyclosporine A (CsA) in the nanomicelles of methoxy poly(ethylene glycol)‐poly(lactide).^[^ ^109^ ^]^ In vivo studies showed the increase in retention effect by 4.5 times compared to 0.05% CsA. This nanomicelle system increased the retention time too, showing a longer effect on dry eye syndrome. Gold‐encapsulated polymeric nanomicelles comprised of copolymer PEG‐*b*‐poly(ɛ‐caprolactone) were prepared by Al Zaki et al. and injected to mice followed by radiotherapy to investigate the deceleration of tumor cell proliferation.^[^ ^110^ ^]^ The improvement was 1.7 times better compared to mice getting radiation therapy only. Belletti et al. loaded curcumin into PLGA nanocarriers which improved the uptake by biological cells whereas uptake of solo curcumin got restricted due to its low bioavailability and low solubility in physiological fluid.^[^ ^111^ ^]^

Natural polymer‐like proteins, for example, albumin, gelatin, polysaccharides, etc., have been broadly developed as matrix‐based nanocarriers for drug delivery because of their fundamental characteristics like biocompatibility, degradability, and easy surface modifications.^[^ ^112^ ^]^ Recently, albumin polymers have been used for preparing drug delivery carriers because of their favorable intrinsic characteristics like several binding locations, highly reacting surface groups, stability, solubility, pH‐responsive nature, nonimmunogenic property, non‐toxicity, and biodegradability.^[^ ^113^ ^]^ Furthermore, they contain larger binding areas and showed 19 days long half‐life. The synergetic effect of cyclosporin A and doxorubicin encapsulated in poly(alkylcyanoacrylate) nanocarriers was studied by Soma et al. and compared with only nanocarriers in resistant tumors.^[^ ^114^ ^]^ It was observed that, this multi‐drug delivery system exhibited better efficiency to inhibit the growth rate as tested in P388/ADR cell line.

These results demonstrate that the use of PNCs in drug delivery can increase the effectiveness of a drug by many folds via drug protection, better solubility, enhanced bioavailability, targeted delivery, etc. which prompts increasing interest of researchers as visible through the growing number of studies (Figure 2a). These PNCs can also exhibit capability to deliver multiple drugs to increase the therapeutic benefits.

**Figure 3:**
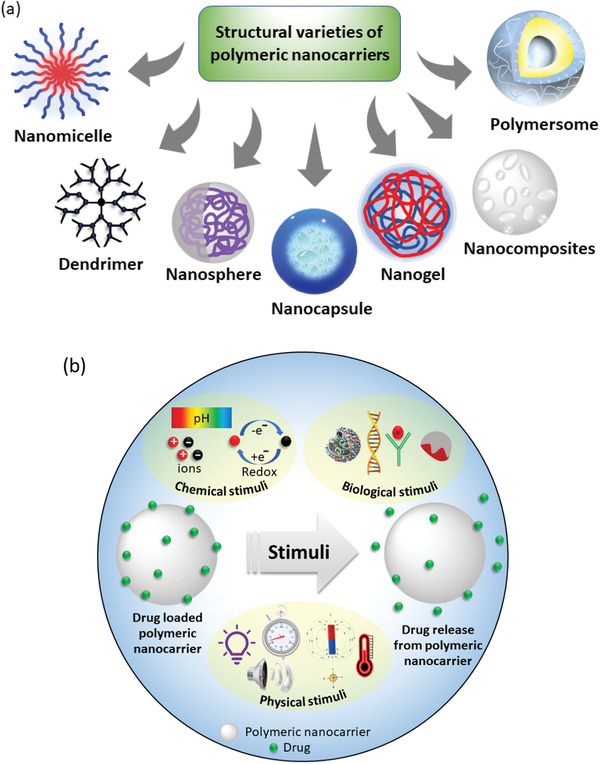
a) Architecture‐based variety of polymeric nanocarriers employed in delivering various types of therapeutics. b) Schematic presentation of drug release from polymer nanoparticle stimulated by various physical, chemical, and biological factors.

Hence, polymeric nanostructured materials have become invaluable for biomedical applications. It is found that the polymers can also be used to design nanocarriers of various architectures (**Figure** **3**a), such as micelles, polymersomes, nanoparticles, nanocapsules, nanogels, dendrimers, and nanocomposites, and be used for therapeutic delivery. They have been widely used in drug delivery, gene therapy, bioimaging, tissue engineering, and regenerative medicine.^[^ ^115^ ^]^ Structures and the functions of these PNCs are elaboratively evaluated in the following sections.

### 2.3 Potential of Stimuli‐Responsive Nanocarriers

A major area of the recent research in drug delivery science is focused on developing stimuli‐responsive nanocarriers by exploiting the availability of various natural, as well as, synthetic polymers which are extremely sensitive to their environmental changes. This is a promising approach of delivering cargo to a specific site at the desired time, that is, the targeted delivery. To design a smart drug delivery system, different stimuli, such as, chemical (e.g., oxidation‐reduction, pH, ion), physical (e.g., temperature, photon, ultrasound), and biological (e.g., enzymes, glucose, inflammation) are being explored (Figure 3b).^[^ ^72^, ^116^, ^117^, ^118^, ^119^, ^120^ ^]^

pH‐responsive polymers, that can accept or donate protons at pathological pH, and thereby undergo moderate conformational changes, are mostly employed for designing these types of carriers.^[^ ^72^, ^118^ ^]^ For example, pH responsive PCL nanoparticles have been used to increase tamoxifen concentrations in estrogen receptor (ER)‐positive breast cancer.^[^ ^121^ ^]^ Luo et al. synthesized amphiphilic stearic acid and carboxymethyl chitosan conjugated self‐assembling nanocarriers loaded with paclitaxel. pH stimulus has also shown effective apoptosis of cancer cells via this platform.^[^ ^122^ ^]^ In a recent study, micelles of PEG conjugated to paclitaxel via disulfide linkage (PEG2000‐S‐S‐PTX) were designed and characterized for its use as a redox‐sensitive prodrug for breast cancer cells.^[^ ^123^ ^]^ The redox‐stimulant drug delivery carriers had promising sensitivity and precision, but the complex biological environment and heterogenetic nature of cancer cells made it challenging to achieve the required specificity of the redox reaction.

Thermosensitive drug delivery carriers require speedy delivery of the encapsulated drug when the tumor microenvironment is at an elevated temperature (≈40–42 °C).^[^ ^94^ ^]^ Production of temperature‐sensitive drug delivery carrier is typically demanding and requires the selection of a polymer that is both safe and responsive to minor changes in temperature around the normal physiological body temperature (37 °C). Other internal stimuli like hypoxia and glucose have also been widely studied for their suitability in nanomedicine formulation.^[^ ^124^, ^125^, ^126^, ^127^ ^]^ Recently, An et al. reported multi‐stimuli responsive PNCs designed by co‐assembling star quaterpolymer with a NIR photothermal agent and a chemotherapeutic compound which exhibited NIR light/pH/reduction‐responsive drug release for cancer treatment.^[^ ^128^ ^]^ Thus, it is foreseeable that the progress of drug delivery science is heading from single‐stimulus responsive to multi‐stimuli responsive carriers due to their single‐point multi‐purpose advantages.

Advances in medical science have also brought in the use of various external stimuli‐based energy sources that efficiently trigger drug release from nanocarriers for effective delivery at the targeted sites. Light, magnetism, ultrasound, and electrical energy are some of the external stimuli being extensively investigated over the recent years.^[^ ^129^, ^130^, ^131^, ^132^, ^133^, ^134^ ^]^

### 2.4 Biodegradable Polymeric Nanocarriers

**Figure 4:**
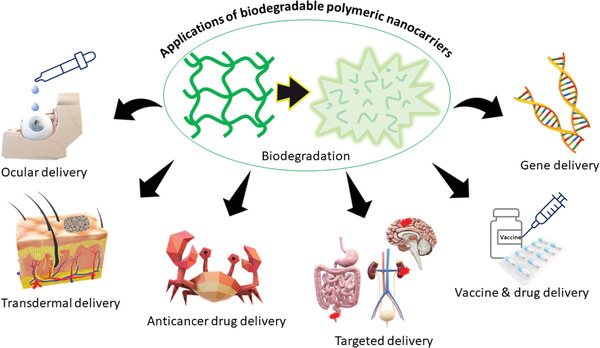
Examples of some advanced applications of biodegradable polymers and their nanostructures in disease treatment.

A concern about the use of non‐biodegradable polymeric nanostructures is that they may lead to problems like chronic toxicity and high immunological response. To minimize these issues and to improve their clearence from the physiological body, use of biodegradable polymeric nanocarriers (BPNCs) are preferred.^[^ ^69^ ^]^ BPNCs are prepared either from preformed polymers or by the polymerization of monomers and are used in bone replacement, surgical materials, plasma expanders and delivery of vaccine, protein, peptide, gene, etc., to treat diabetes, various cancers, cerebral, ophthalmic, inflammatory, and infectious diseases.^[^ ^135^, ^136^, ^137^ ^]^ There has been great potential of BPNCs in medical applications as they can play significant role in diagnosis for the treatment of different types of diseases, in medical imaging, biomarkers, biosensors, nanomachines, nano‐robots, and nano drug delivery systems low cytotoxicity.^[^ ^119^, ^138^ ^]^ Due to the biodegradability, BPNCs are superior to conventional drug carriers, which are often orally administered in a patient in either capsule or liquid forms. BPNCs are being chemically engineered to develop highly selective and stable properties for advanced applications (**Figure** **4**).^[^ ^139^, ^140^ ^]^ Examples of some biodegradable polymers used for designing PNCs are polyesters, polyamides, polysaccharides, proteins, polyphosphorous, and polyanhydrides.^[^ ^140^ ^]^ The major research challenge in the field of BPNCs is to develop appropriate surface and functional properties which are essential for synthetic reproducibility, scale‐up procedures, in vivo assessment, tracking, and imaging. The use of BPNs in drug delivery enables passive and active targeting as carriers, like a vehicle, to inflamed and diseased tissues with increased vascular leakiness, overexpression of specific epitopes, and cellular uptake.^[^ ^141^, ^142^ ^]^ BPNs have been extensively advocated as particulate carriers in the pharmaceutical and medical fields because of their promising roles as drug delivery systems, their controlled and sustained release properties, subcellular size, and biocompatibility with tissues. However, some recent studies have indicated that even the biodegradable polymer, such as, PLGA, can demonstrate toxicological profile.^[^ ^143^, ^144^, ^145^ ^]^ This is because the physicochemical properties of the polymers are susceptible to alteration in their nanoformulations due to high surface area to mass ratios of nanostructures.^[^ ^146^ ^]^ Xion et al. have demonstrated how the size influenced cytotoxicity in both RAW264.7 cells and BEAS‐2B cells.^[^ ^147^ ^]^ Recently, Grabowski et al. have reported that with the increase in concentration of PLGA nanoformulation, the production of reactive oxidative stress increased in co‐cultured lung epithelial cells and human‐like macrophages.^[^ ^148^ ^]^ This group has further found that different PLGA formulations have also exhibited mild inflammation.^[^ ^149^ ^]^ Sing and Ramarao have also reported similar outcome.^[^ ^150^ ^]^ A study by Guo et al. have shown that the drug loaded PEG‐PLL‐PLGA (PLL: poly‐L‐lysin) nanocarriers have shown feeble toxicity in Kunming mice but the results for blank nanocarriers are not available, so the origin of toxicity could not be confirmed.^[^ ^151^ ^]^ Semete et al. have carried out a comparison study where toxicity caused by silica‐, zinc‐, and iron‐based nanoparticles to that of PLGA nanocarriers and found that there was no appreciable toxicity shown by the PNCs as it was shown by the inorganic nanoparticles.^[^ ^152^ ^]^ Recently, a comparitive study on the influence of two different shapes of PLGA‐PEG (PEG: polyethylene glycol) nanoparticles were investigated where it was found that the needle‐like shape showed higher toxicity than the spherical particles.^[^ ^153^ ^]^ More investigations on the immune response of BPNCs are required as such information are important but not yet well documented.

### 2.5 Section Summary

PNCs are the self‐assembly of polymer molecules capable of drug loading, protecting, and delivery. There are various types of structurally different PNCs in use, namely, nanomicelle, nanogel, nanosphere, nanocapsules, dendrimers, etc. Use of these nanostructures for therapeutic transport multiplies the success of a drug by many folds. They can load not only small drug molecules but also macromolecular therapeutics. These nanocarriers extend the lifetime of drugs in circulatory system, facilitate drug passage through various microtubules having tiny orifices, and reach the targeted sites even to the organelles. Nanocarriers composed of various stimuli‐responsive polymers are being most popularly used. These stimuli can be chemical, physical, and/or biological. Among various types of polymers, the most desired ones are the polymers that are biodegradable, as well as, stimuli responsive.

## 3 Various Polymeric Nanocarriers for Drug Delivery

The solid colloidal particles which are generally of 10 nm to the highest size limit of 1000 nm, are generally termed as nanoparticles in various literatures.^[^ ^154^ ^]^ However, here we have assessed the polymeric carriers whose dimensions are within 200 nm. Nanospheres, nanorods, nanostars, nanocapsules, etc., are collectively known as nanoparticles. Polymer nanoparticles have a solid matrix, and the drug molecules are either loaded on the surface or encapsulated within the particles. In general, a nanosphere is solid sphere and mostly adsorbs therapeutics on its surfaces while nanocapsules act as a reservoir which encapsulates substances that are bound to a cavity of either lipid or water core.^[^ ^155^ ^]^ Nanorods and nanostars are also in use as nanocarriers however the complexity of designing these structures has kept them away from being widely used.

Paul Ehrlich started the development of polymer nanoparticles and the first experiment was conducted by Ursula Scheffel, while in late “60s and ”70s a large number of works were carried out by Peter Speiser, et al.^[^ ^156^, ^157^, ^158^ ^]^ Since then, PNCs have been widely employed in designing various biomedicines. Compared to other drug carriers, nanoparticles are stable and tight, and can be easily prepared and piloted. Drug‐loaded nanoparticles have been applied for subcutaneous or intravenous injection, oral administration, and so on.

As drug carriers, PNCs have shown several advantages, such as, good stability, higher therapeutic loading efficiency, smooth intracellular uptake, and biocompatibility. More importantly, PNCs can be engineered for targeted delivery to increase the rate of success in disease treatment and decrease the side effects of drugs.^[^ ^156^, ^159^ ^]^ Despite of these advantages, PNCs still have various hurdles to overcome. In many of the cases they are fragile, non‐biodegradable, expensive to manufacture and toxic solvent residuals are often present. Complete removal of organic solvent residuals is tedious and costly. Thus, minimizing toxicity associated with PNCs has always been challenging.^[^ ^160^ ^]^ Assessments of some popularly used PNCs are presented in the following sections.

### 3.1 Nanomicelles

**Figure 5:**
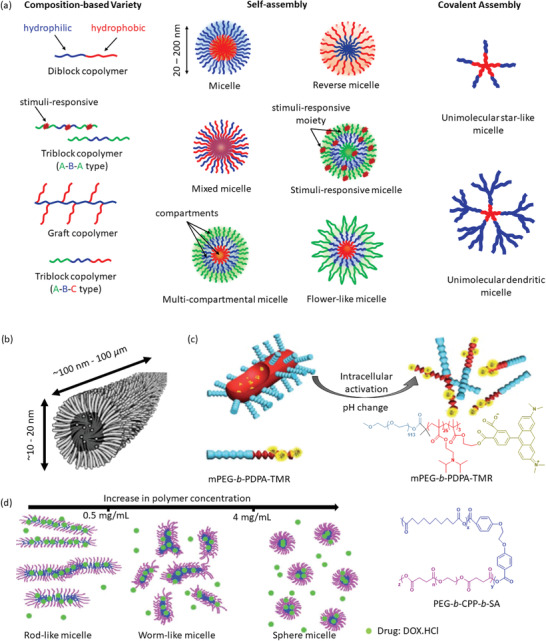
a) Various polymeric nanomicelles that can be fabricated depending on the architectures of copolymers and inter‐chain interactions. b) Schematic presentation of a wormlike micelle having hydrophobic core surrounded by hydrophilic blocks of amphiphilic polymers. Reproduced with permission.^[^ ^167^ ^]^ Copyright 2003, American Chemical Society. c) Intracellular pH‐activated drug release from wormlike micelle composed of dye‐tagged diblock copolymer poly(ethylene glycol)‐*b*‐poly(2‐diisopropyl methacrylate)‐tetramethyl rhodamine (mPEG‐*b*‐PDPA‐TMR). Reproduced with permission.^[^ ^168^ ^]^ Copyright 2010, Royal Society of Chemistry. d) Concentration dependent self‐assembly mechanism of micelle formation composed of triblock biodegradable copolymer poly(ethylene glycol)‐*b*‐1,3‐bis(*p*‐carboxyphenoxy) propane‐*b*‐sebacic acid (PEG‐*b*‐CPP‐*b*‐SA). Abbreviation. DOX.HCl: Doxorubicin hydrochloride. Reproduced with permissions.^[^ ^169^ ^]^ Copyright 2005, Royal Society of Chemistry.

The most common and stable nanostructures of amphiphilic macromolecules in aqueous media are polymeric micelles and vesicles (polymersomes).^[^ ^72^, ^161^ ^]^ Various types of polymeric micelles can be obtained via the self‐assembly of amphiphilic copolymers and are broadly studied for drug delivery.^[^ ^162^ ^]^ Depending on the composiiton of copolymer and inter‐chain interactions, various types of micelles can be designed. In general, these micelles may consist of a hydrophobic core and hydrophilic corona, whereas in a reverse micelle this arrangement can be switched to hydrophilic core and hydrophobic corona. Thus, both the hydrophilic and hydrophobic drugs can be loaded into these polymeric micelles. Again, a single micelle can simultaneously load hydrophilic and hydrophobic drugs engaging both the corona and core, resulting in the formation of multidrug delivery polymeric micelle. Exploiting the inter‐molecular interactions, these micelles can also be designed as mixed, flower‐like, multicompartmental, star‐like, and dendritic micelles (**Figure** **5**a). Their sizes may vary from 20 to 200 nm.^[^ ^163^ ^]^ Polymeric micelles have low critical micelle concentration (CMC) than that of surfactants making them comparatively more stable and thus are extra advantageous in carrier design for biomedicine development. This implies that less polymeric materials will be required to prepare a micelle in comparison to that of surfactants. Yang et al. prepared a micelle type polymeric drug delivery carrier based on pH‐sensitive poly(poly‐(ethylene glycol) methyl ether monomethacrylate micelles prepared for the oral administration of hydrophobic drugs.^[^ ^120^ ^]^ The CMC values are quite low and ranged from 1.4 to 2.6 mg L^–1^ and the average hydrodynamic sizes were 140–250 nm as determined by dynamic light scattering technique. This has been applied for the delivery of a hydrophobic drug nifedipine which achieved ≈96% release at physiological pH 7.4 within 24 h.

Loading of drugs can be achieved either by physically entrapping them within the core of a micelle or it could also be the polymer‐drug conjugates that are pre‐formed prior to their micellization.^[^ ^164^ ^]^ Encapsulation of drug within a micelle significantly increases solubility and bioavailability of the pharmaceutically active ingredient. It also decreases the toxic side effects of drugs leaving untargeted organs and tissues safe.^[^ ^165^ ^]^ Micelles can also augment the lifetime of drugs.^[^ ^30^ ^]^ The disadvantages of polymeric micelles are their low stability when diluted in physiological fluids and sensitivity to increased ionic strength. These both often cause premature release of drugs.^[^ ^166^ ^]^ Self‐aggregation of amphiphilic copolymers can also lead to long rod‐like structure, whose diameter ranges in nanometer scale while the length can grow to be of a micrometer, known as polymeric wormlike micelles (Figure 5b).^[^ ^167^ ^]^ These wormlike micelles are better capable of penetrating nanoporous gels, due to their shape, compared to small‐sized vesicles or spherical particles composed of similar materials. Yu et al. have demonstrated that the wormlike micelles can also deliver drugs activated by their environmental pH change (Figure 5c).^[^ ^168^ ^]^ The micelle composed of poly(ethylene glycol)‐*b*‐poly(2‐diisopropyl methacrylate) remained “silent” at physiological pH (≈7.4) but got activated in the acidic environment of endocytic vesicles (pH ≈ 5–4). The morphologies of wormlike micelles can be altered by varying concentration and architecture of component polymers as demonstrated by Chen et al. which is summarized in Figure 5d.^[^ ^169^ ^]^ Thus, wormlike micelles can undergo morphological transition influenced by external stimuli, such as, pH, heat, carbon dioxide, redox, solvent, etc.^[^ ^170^ ^]^

### 3.2 Nanovesicles

**Figure 6:**
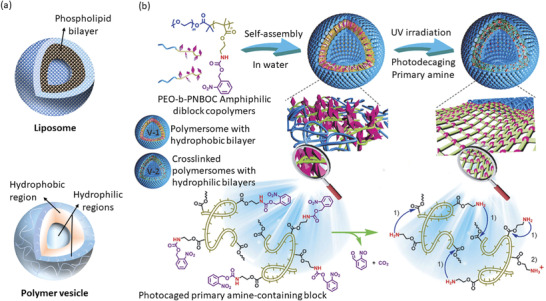
a) Schematic presentation of liposome and polymer nanovesicle. b) A novel photo‐responsive polymersome capable of co‐loading both the hydrophilic and hydrophobic drugs. Reproduced with permission.^[^ ^176^ ^]^ Copyright 2014, Wiley‐VCH Verlag GmbH & Co. KGaA, Weinheim.

Polymeric vesicles, also known as polymersomes, are PNCs that have a bilayer membrane composed of a hydrophobic layer trapped between hydrophilic core and hydrophilic shell (**Figure** **6**a).^[^ ^171^ ^]^ These are constituted by amphiphilic copolymers and have found numerous biomedical applications including programmed drug delivery for cancer therapy.^[^ ^161^, ^172^, ^173^ ^]^ The first scientific study was performed by Discher et al. in the year 1999 and coined the term polymersomes which is in resemblance with liposomes.^[^ ^174^ ^]^ The size of vesicles plays important roles in regulating circulation time, RES recognition, biodistribution, and process of cellular uptake. Considering these processes, the optimum sizes of vesicles are expected to be in the range of 80–150 nm.^[^ ^175^ ^]^ Nanovesicles can encapsulate hydrophilic molecules within the aqueous interior which is isolated from the external medium and can simultaneously integrate hydrophobic molecules within the membrane. Hence, these vesicles can co‐deliver both the hydrophilic and hydrophobic drugs, such as, anticancer therapeutics, genes, and proteins. Wang et al. have prepared a novel photo‐responsive polymersome capable of co‐loading a hydrophilic anticancer drug doxorubicin hydrochloride and a hydrophobic model drug Nile red via self‐assembly (Figure 6b).^[^ ^176^ ^]^ Thus, these nanovesicle PNCs have huge potential to be explored further for popularizing as multi‐drug delivery nanocarriers.

In comparison to the molecular weights of lipids in liposomes, polymersomes can have their component copolymers of higher molecular weights, thus can demonstrate better physical and chemical stability.^[^ ^177^ ^]^ The molecular weights of copolymers can be up to 100 kDa, while that of lipid is usually less than 1 kDa. A schematic presentation of liposome and polymer vesicle is presented in Figure 6a showing their structural similarities. Physicochemical properties of polymersomes can be altered by the judicial choice of appropriate polymer, correct molecular weights, and ratio of hydrophobic to hydrophilic segments.

These nanocarrier‐based materials are also being increasingly investigated to combat coronavirus disease 2019 (COVID‐19), a disease caused by infection with severe acute respiratory syndrome coronavirus 2 (SARS‐CoV‐2).^[^ ^178^ ^]^ Presently, there are about 26 nanocarrier‐based vaccines being developed for COVID‐19 treatment which are under human clinical trials while about 60 more candidates are at different stages of development.^[^ ^179^ ^]^ Nanocarrier‐based vaccines are advantageous as their structural features can be designed to mimic a virus and their sizes can be tuned to the 100–200 nm range.^[^ ^180^ ^]^ Two main strategies of loading vaccine antigens or nucleic acid cargoes onto the nanovesicles are: i) Loading on the surface, and ii) encapsulation at the core. Having these cargoes loaded on the nanocarrier surface can lead to potent immunogenicity, while the encapsulated active agents at the core can stay protected and thereby demonstrate controlled delivery.^[^ ^179^ ^]^ The first two vaccines approved for treating COVID‐19 are comprised of lipid nanocarriers with encapsulate mRNA encoding engineered spike (S) protein as active material.^[^ ^181^, ^182^ ^]^ However, polymers such as, PLGA, polyethyleneimine, chitosan, etc., have been widely used to develop nanocarriers to deliver vaccines for various other corona viruses.^[^ ^178^ ^]^ Recently, Renu et al. have designed chitosan‐based PNC to encapsulate SARS‐CoV‐2 receptor‐binding domain protein.^[^ ^183^ ^]^ They have demonstrated that upon intranasal administration, the positive surface charge of chitosan‐based nanocarriers can electrostatically interact with the negatively charged sialic acid and thereby facilitate the adhesion of vaccine nanocarriers onto the epithelial surfaces of airways which is the actual route of the virus. Here the surface of nanocarriers have been modified to facilitate their transport to the target cells. These PNCs can also be engineered to open the tight junctions of mucosal barriers and facilitate the transport when a vaccine is administered intranasally mimicking the pathway of a viral infection such as, the path of SARS‐CoV‐2. Thus, polymer nanovesicles can be promising candidates for vaccine delivery to combat COVID‐19. To design such nanovesicular PNCs, lipophilic/amphiphilic polymers can be employed as potential candidate components since they can form the nanovesicles mimicking liposomes. These polymers can be synthesized to possess lipid like properties,^[^ ^184^ ^]^ desired chain length (molecular weight) to achieve optimum physicochemical stability, and finally they can form liposome‐like vesicles of nanodimension whose surfaces can be modified to demonstrate target cell recognition properties and stimuli‐responsive behavior while its core can safeguard the cargoes.^[^ ^171^ ^]^ Lin et al. have designed hollow PNC having aqueous core and PLGA‐based shell to encapsulate soluble viromimetic stimulator of interferon genes adjuvant for vaccine delivery against the middle east respiratory syndrome coronavirus.^[^ ^185^ ^]^ PLGA is pH responsive and hence can readily release the adjuvants once inside the cytosol. Nanovesicular vaccines have additional advantage that their size, charge, and shape can readily be tuned. They can be prepared as responsive to multiple stimuli (e.g., pH, cell, antibody, etc.) and co‐carry multiple loads (e.g., targeting moieties, adjuvants, etc.). Furthermore, the component polymer molecules can be ionizable and bear cationic charges at low pH while at physiological pH they can remain neutral. Hence, such molecules can facilitate the loading of anionic mRNA molecules at low pH via the electrostatic interactions. These loaded nanocarriers can be endocytosed by cells, get positively charged in the low pH environment of endosomes, and thereby disrupt the endosomal membrane resulting release of mRNA into the cytoplasm, where the mRNA can be translated to protein. Use of mucoadhesive polymers in designing these nanovesicle PNCs can also allow the formulations to be used for intranasal administration to follow the actual route of virus as is the case of COVID‐19. However, the loading efficacy of especially the large proteinaceous cargos into these PNCs are still to be improved. At present all the 22 COVID‐19 vaccines being used worldwide have incorporated S protein or its derivatives. To achieve efficient delivery of mRNA, lipid‐polymer hybrid PNCs have also shown promising results.^[^ ^186^ ^]^ The intermolecular noncovalent interactions between the component polymer molecules facilitate nanocarrier formation and stabilization. Controlled PEGylation of PNCs can also minimize the chance of opsonization in vivo. Nucleic acids, such as, DNA, RNA, etc., encoding viral antigens can be delivered by encapsulating them within polymeric nanovesicles which also contributes to their protection from easy degradation often caused by RNases.^[^ ^187^, ^188^ ^]^ These pioneering studies have demonstrated that the nanovesicular PNCs have the advantages of biocompatibility and biodegradability, size tuning, co‐loading of adjuvants and active agents, colloidal stability, stimuli‐responsive properties, and antigen functionality. Thus, polymeric nanovesicles are one of the potential multi‐stimuli responsive multi‐drug delivery nanocarrier which can become a prominent candidate to advance vaccine research and provide solution to the ongoing pandemic and beyond.

From the initial simple hollow spheres, vesicles are now developed into much sophisticated bio‐inspired nanostructures, such as, the multi‐compartment vesicles. They are developed as stimuli‐responsive and have wide applications as nano‐reactors, biosensors, catalyst, biomedicine, etc. Despite these advantages, they have some limitations, too. They often show low entrapping efficiency.^[^ ^72^ ^]^ The monomer of vesicles should be biocompatible and biodegradable. The vesicles should have multiple functionalities and require a suitable synthesis process that can support mass production.^[^ ^189^ ^]^

### 3.3 Nanogels

**Figure 7:**
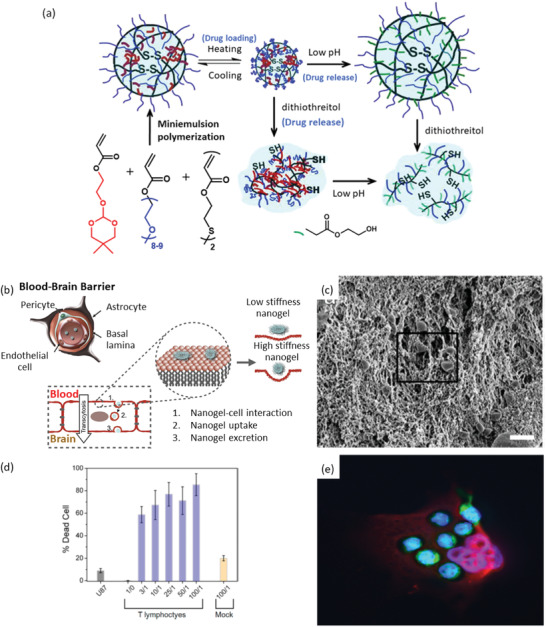
a) A schematic presentation of nanogel synthesis by miniemulsion polymerization and its pH‐responsive drug release phenomena for cancer therapy. Reproduced with permission.^[^ ^195^ ^]^ Copyright 2011, Elsevier. b) Nanogel transcytosis across an in vitro blood‐brain barrier facilitated by low nanogel stiffness. Reproduced under the terms of a Creative Commons Attribution 4.0 International License.^[^ ^204^ ^]^ Copyright 2021, The Authors. ublished by Elsevier. c) The scanning electron microscopic image of T lymphocyte invading through the poly(ethylene glycol)‐g‐chitosan gel. d) Percent of tumor cell death (U‐87 MG cell line) caused by T lymphocyte treatment at various T lymphocyte/U87 ratios (1/0, 3/1, 100/1). Mock is the negative control. e) Fluorescence image of T lymphocytes (green) attached to U‐87 MG cells (red) after crossing through the transwell membrane mimicking the blood‐brain barrier under in vitro test condition. c–e) Reproduced with permission.^[^ ^207^ ^]^ Copyright 2014, American Chemical Society.

In recent years, nanogels have got popularized due to their high loading capacity and improved stability.^[^ ^116^ ^]^ They are polymeric 3D nanostructures, and their properties can be tailored. These nanogels can be designed in various structures, such as, simple nanogel sphere, core‐shell, hollow, functionalized, hairy, multilayer, etc.^[^ ^190^ ^]^ Polymeric nanogels are swellable nanosized hydrogel dispersions fabricated by chemical or physical crosslinking of hydrophilic or amphiphilic polymer chains.^[^ ^191^, ^192^ ^]^ Nanogels can be prepared as stimuli‐responsive for drug delivery. They are responsive to temperature, pH, light, magnetic field, biomolecule recognition, etc.^[^ ^193^, ^194^ ^]^ Since, pH and temperature are often common stimuli in both the biological, as well as, chemical environment, these nanogels have been prepared as dual‐ or even multi‐stimuli responsive. Qiao et al. have designed multi‐responsive nanogel as carriers for hydrophobic anti‐cancer drugs, for example, Nile Red, paclitaxel, and doxorubicin, and have studied their release behavior (**Figure** **7**a).^[^ ^195^ ^]^ The carrier exhibited acid‐triggered hydrolysis, thermo‐responsive performance, and degradation induced by reduction. The unloaded nanogel was found to be nontoxic presenting the carrier as promising candidate for various other hydrophobic anticancer drugs.

Nanogels are often non‐cytotoxic, although sometimes it is dose and time dependent phenomenon. Temperature sensitive poly(*N*‐vinylprolactam)‐based nanogels were found to be non‐cytotoxic to both 9‐day and 16‐day neurons from the cerebral cortex of rat embryos for up to 24 h based on the outcome of MTT(3‐(4,5‐Dimethylthiazol 2‐yl)‐2,5‐diphenyltetrazolium bromide) and LDH (lactatae dehydrogenase) assays.^[^ ^196^ ^]^

Conjugation of nanogels to biomaterials are often applied to modulate their biocompatibility. Khan et al. developed gold nanorod‐nanogel (Au NRs‐nanogels) composite particles by connecting gold nanorods to poly(*N*‐isopropyl acrylamide)‐based nanogels via electrostatic interactions.^[^ ^197^ ^]^ The Au NRs‐nanogels showed reduced cytotoxicity to MCF‐7 cells, human breast cancer cell line based on the results obtained in MTT and LDH assays. In addition, the nanogel almost eliminated the hemolytic activity of the gold nanorods on blood agar.

Ketoprofen, an anti‐inflammatory drug, inhibits cyclooxygenase and shows high gastrointestinal toxicity. Encapsulation by nanogels based on cellulose acetate phthalate and hydroxyethyl methacrylate (CAP‐*co*‐poly (HEMA)), reduced the cytotoxicity of ketoprofen against Vero cells, which are monkey kidney epithelial cells, after 24 h of incubation at a concentration range of 1–20 µg mL^–1^.^[^ ^198^ ^]^ 2‐hydroxy‐1‐(4‐(2‐hydroxyethoxy)phenyl)‐2‐methyl‐1‐propanone (Irgacure 2959) is an often used photo‐initiator for water borne photo‐curing of biomaterials because of its excellent water solubility and low toxicity which has shown effective results in designing hydrophilic nanogels with uniform size distribution and improved biocompatibility.^[^ ^199^ ^]^ Recently, polymer nanogels have been presented as next‐generation smart nanocarrier. Here, drug was conjugated to nanogel matrix through H‐bond.^[^ ^200^ ^]^ Supramolecular polymer nanogel fabricated via host‐guest interaction has exhibited enhanced penetration during dermal drug delivery.^[^ ^201^ ^]^ Chen et al. have prepared multi‐responsive polymer nanogel with self‐healing property which has good potential for biomedical engineering as it responded to pH, hydrogen peroxide, sugar, adenosine triphosphate, and temperature.^[^ ^202^ ^]^ Polymer nanogel of size 136 ± 37.6 composed of bio‐derived anionic poly(*γ*‐glutamic acid) has demonstrated excellent biocompatibility and controlled release of anticancer drug doxorubicin.^[^ ^203^ ^]^ Recent development in polymeric nanogel is also showing promising results in brain cancer treatment. It is known that most of the therapeutics (molecular weight greater than 500 Da) cannot pass through BBB resulting in it a very challenging to treat neurodegenerative diseases. Through an in vitro experiment, Ribovski et al. have demonstrated that nanogels can be a promising candidate to achieve permeation through BBB due to their low stiffness (Figure 7b).^[^ ^204^ ^]^ For treating brain tumor, hydrogels composed of poly(ethylene glycol)‐*g*‐chitosan (PCgel) and containing T lymphocytes has been used to achieve localized delivery at the glioblastoma cells (Figure 7c–e).^[^ ^205^, ^206^, ^207^ ^]^ Thus, this PCgel can be a promising emerging candidate for localized immunotherapy for glioblastoma, an aggressive cancer.

**Figure 8:**
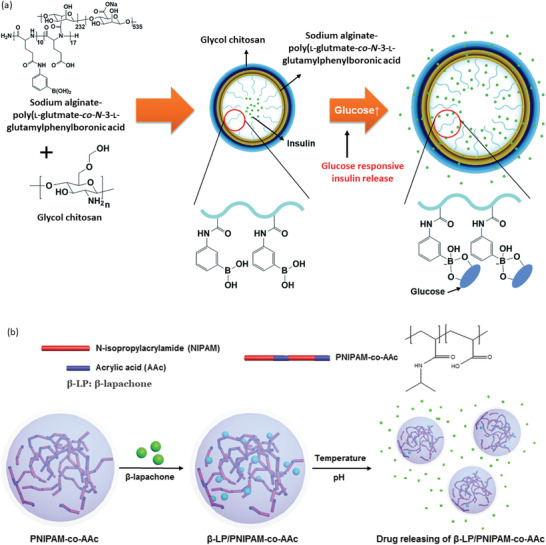
a) Schematic presentation of controlled insulin release from polymeric nanogel triggered by the presence of glucose. Reproduced with permission.^[^ ^208^ ^]^ Copyright 2015, Royal Society of Chemistry. b) Poly‐*N*‐isopropyl acrylamide‐based nanogel as double‐stimuli‐responsive (temperature and pH) drug carrier. Reproduced under the terms of a Creative Commons Attribution 4.0 International License.^[^ ^209^ ^]^ Copyright 2019, The Authors. Published by Springer Nature.

To have improved control on insulin release, Lee et al. have designed glucose‐responsive nanogel which also showed better biocompatibility and biostability (**Figure** **8**a).^[^ ^208^ ^]^ The carrier is a glycol chitosan (GC)/sodium alginate (SA)‐poly(L‐glutmate‐*co*‐N‐3‐L‐glutamylphenylboronic acid) (PGGA) graft polymer‐based double‐layered nanogel, synthesized by *N*‐carboxyanhydride polymerization and carbodiimide coupling reactions. The in vitro study showed that the carrier released insulin at the diabetic glucose level. It is always desirable that along with controlled release, a carrier can be multi‐triggered which is a promising outcome. Recently, Kim et al. have designed poly‐*N*‐isopropyl acrylamide nanogels modified by acrylic acid which responded to both the temperature and pH (Figure 8b).^[^ ^209^ ^]^ Thus, these works have demonstrated that polymer‐based nanogels having the pH‐, temperature‐, glucose‐, etc., responsive features can be the way forward to design multi‐stimuli‐responsive carriers.

### 3.4 Nanocapsules

**Figure 9:**
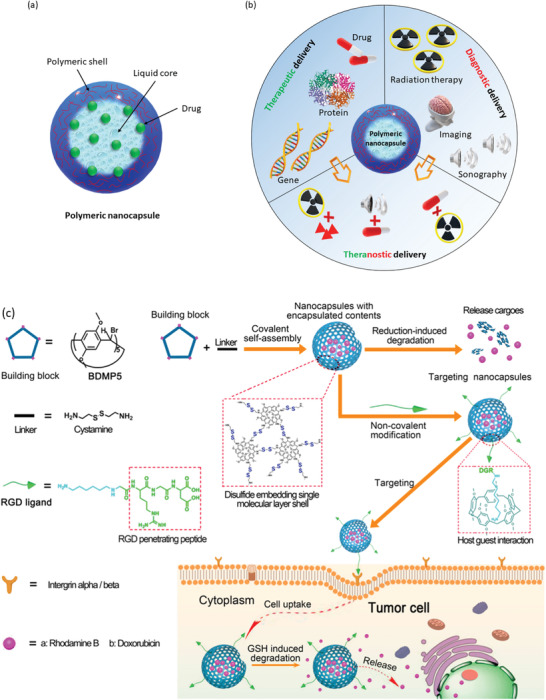
a) Nanocapsule having polymeric shell and liquid core with encapsulated drug molecules. b) Schematic illustration of polymeric nanocapsules and their use in various bioapplications.^[^ ^220^ ^]^ Protein image (1HFW) is from protein data bank (https://www.rcsb.org/structure/1HFW). c) Design strategy and anticancer drug delivery mechanism of reducing agent glutathione responsive pillar[5]arene‐based single molecular layer polymer nanocapsule demonstrating the targeted delivery. Reproduced with permission.^[^ ^221^ ^]^ Copyright 2018, American Chemical Society.

Nanocapsules are hollow spherical structures which can be prepared by miniemulsion polymerization, using sacrificial templates, and nanoprecipitation.^[^ ^210^, ^211^, ^212^ ^]^ Typical nanocapsules are composed of a cross‐linked polymer shell and a hollow inner space containing liquid, for example, lipid (**Figure** **9**a). Drugs are confined in the cavities of nanocapsules and surrounded by external polymer membranes. Kahattab et al. have shown that nanocapsules can improve the oral bioavailability of proteins and peptides.^[^ ^213^ ^]^ Thus, nanocapsules can prohibit degradation of drugs, reduce systemic toxicity on normal cells, provide controlled release, and mask unpleasant taste.^[^ ^214^ ^]^

Marto et al. reported nanocapsule having oily core modified with shell made of starch. The capsules were synthesized via the emulsion‐solvent evaporation method. The developed nanocapsules showed good stability and with no irritation or tolerability issues faced during topical administration.^[^ ^215^ ^]^ This research group has further developed another nanocapsule based on modified starch containing minocycline hydrochloride following a similar synthesis method. The process is optimized through a factorial design which produced a particle size distribution ≈90 nm and the resulting capsules showed an encapsulation efficiency of over 87%.^[^ ^216^ ^]^ For the treatment of alopecia, Lee et al. developed a nanocapsule system comprised of chitosan and Pluronic F127 fabricated via nanoprecipitation technique. The fabricated nanocapsule exhibited an encapsulation efficiency up to 5% of the drug cyclosporine A for the systems with a median size below 100 nm. Chitosan nanocapsule formulation showed an improvement of cyclosporine A absorption by mouse skin resulting an increase in the number of hair follicles.^[^ ^217^ ^]^ For the targeted administration of proteins at the colon level, Zhang et al. synthesized nanocapsules through layer‐by‐layer technique using different modified starches bearing opposing ionic charges. The study suggests that an optimal system for the controlled release of proteins at the colon level can be synthesized by carefully selecting the function parameters (degree of substitution and molar mass) of the employed polymers.^[^ ^218^ ^]^ Recently, Dubey et al. develop chitosan and pectin nanocapsules for ocular delivery of brinzolamide for the treatment of glaucoma. The carrier was synthesized through coacervation technique and the obtained nanocapsules have sizes in the range of 217.01 ± 0.21 nm to 240.05 ± 0.08 nm. During the in vitro studies, the system showed superior release profile compared to a commercial drug product containing brinzolamide suspension. Ex vivo studies showed an increased residence time of the nanocapsule formulation at the substrate level, and a better penetration at the superior cornea level, more efficiently reducing the intraocular pressure compared to the marketed drug formulation.^[^ ^219^ ^]^ These nanocapsules are being not only prospective for therapeutic delivery, but also for theranostic and diagnostic delivery (Figure 9b).^[^ ^220^ ^]^ Fu et al. have designed a smart polymer nanocapsule having reductive‐responsive pillar[5]‐ arene‐based single‐molecule‐layer which showed excellent uptake by targeted tumor cells (Figure 9c).^[^ ^221^ ^]^ The system showed good biocompatibility and triggered release of encapsulated into the intracellular space of tumor cells having high concentration of reducing agent glutathione (GSH). Here, the degradation of the nanocapsule and drug release were induced by the GSH resulting this carrier to achieve highly targeted delivery.

### 3.5 Dendrimers

Dendrimers (or dendritic polymers) are 3D, highly ordered star‐like oligomeric and polymeric macromolecules.^[^ ^115^, ^222^ ^]^ The sizes of dendritic nanocarriers often vary from 5 to 10 nm which is very much favorable for pulmonary and intravenous system.^[^ ^223^ ^]^ The variations in architectures of different types of dendrimers evolve based on the molecular chirality, composition and branching of the initial generation (G0) of the molecule used, that is, the molecule at the core. Because of their numerous exploitable properties, such as, nano dimension, narrow polydispersity index, excellent control over molecular structures, hyperbranched polymeric composition, different void volumes, multivalency, high biocompatibility, dendrimers are gaining increasing interests in designing nanomedicines.^[^ ^211^, ^224^ ^]^ Thus, polymeric dendrimers are being progressively used in the delivery of bioactive agents such as drugs, oligonucleotides, enzymes, vaccines, and genes.^[^ ^225^, ^226^, ^227^, ^228^, ^229^ ^]^ They have also been found to be suitable for drug‐specific, as well as, site‐specific nanocarrier design in anticancer therapy.^[^ ^230^, ^231^ ^]^

**Figure 10:**
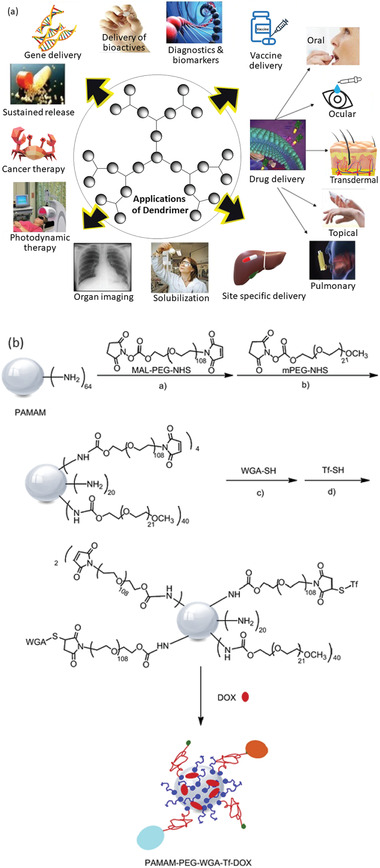
a) Vast application field of dendrimer‐based nanocarriers in drug delivery. Reproduced with permission.^[^ ^222^ ^]^ Copyright 2013, Elsevier. b) The synthetic route of dual‐targeting drug carrier polyamidoamine‐poly(ethylene glycol)‐wheat germ agglutinin‐transferrin‐doxorubicin (PAMAM‐PEG‐WGA‐Tf‐DOX). Reproduced with permission.^[^ ^243^ ^]^ Copyright 2010, Elsevier.

**Figure 11:**
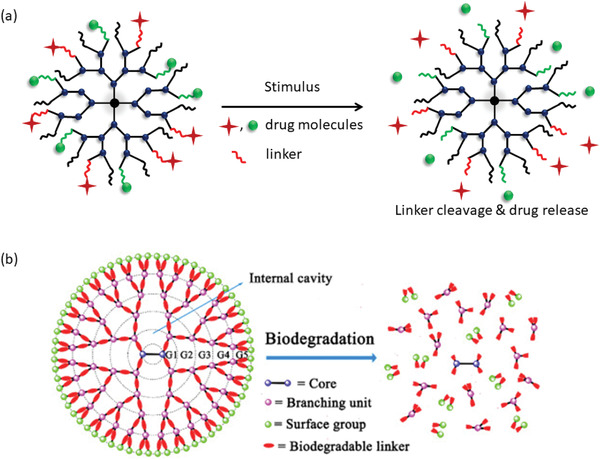
a) Stimuli‐responsive in vivo degradation of dendrimer. b) Schematic presentation of biodegradation of a biodegradable dendrimer. Reproduced with permission.^[^ ^245^ ^]^ Copyright 2018, Elsevier.

As most of the therapeutics suffer from drug resistance, toxicity, insolubility, unfavorable administrative routes and biological barriers, dendrimer‐based drug delivery carriers have been used to transport cargos to the targeted cells with much reduced toxicity during a pharmaceutical application.^[^ ^232^ ^]^ Polyamidoamine (PAMAM) dendrimers, consisting of ethylenediamine core and branching units of amine groups, have been utilized to load enzymes, antibodies, drugs and other bioactive molecules as delivery carrier owing to their non‐ immunogenic, hydrophilic and biocompatible nature.^[^ ^233^, ^234^, ^235^ ^]^ Poly‐L‐lysine (PLL) and poly(propylene imine) (PPI) dendrimers consisting of two primary amines modified to enhance therapeutic actions, and ethylenediamine or 1,4‐diaminobutane having the branching units of propylene imine monomers respectively, are mostly utilized as gene delivery carriers to the targeted sites owing to their favorable characteristics.^[^ ^236^ ^]^ In this context, the formed PPI dendrimer–DNA complex is endocytosed into the cells followed by endosome destabilization of these electrostatically assembled complex resulting subsequent DNA release to the targeted cells.^[^ ^237^, ^238^ ^]^ It is demonstrated that the anticancer drug cisplatin encapsulated within PAMAM dendrimers exhibits low toxicity, gradual release and higher uptake in solid tumors in comparison to free cisplatin, while carboxylate terminated PAMAM dendrimers loaded with this drug showed higher efficacy in tumor bearing mice.^[^ ^239^ ^]^ The antitumor and antimicrobial activities of dendritic polymer cargos and silver salts encapsulated in PAMAM dendrimers are also noteworthy.^[^ ^239^, ^240^ ^]^ Recent research outcomes have also demonstrated folate anchored PLL or PPI dendrimers as doxorubicin hydrochloride (DOX.HCl) or methotrexate (MTX) nanocarriers against selective targeting of cancer cells to achieve improved efficiency as anticancer agent.^[^ ^241^, ^242^ ^]^ Thus, dendrimer‐based nanocarriers have found vast application field in drug delivery carrier design as summarized in **Figure** **10**a.^[^ ^222^ ^]^ These dendrimer‐based nanocarriers have also been successfully used for dual drug delivery purposes, for example, delivery of gene‐based medicine and cancer drug. They have also been successful in delivering dual drugs across the BBB to treat brain tumor. In a study, He et al. presented PEGylated G4 (Generation 4) PAMAM dendrimer nanocarrier (14–20 nm) modified with both the transferrin (Tf) and wheat germ agglutinin (WGA), the brain‐targeting ligands, loaded with doxorubicin at the dendrimer interior which served as dual targeting drug carrier.^[^ ^243^ ^]^ The study showed that the carrier (PAMAM‐PEG‐WGA‐Tf) delivered 13.5% of doxorubicin across the BBB within 2 h which is much higher compared to free doxorubicin (5%), and PAMAM‐PEG‐Tf (7%) (Figure 10b). Like various other nanocarriers, dendrimer‐based nanocarriers can also be stimuli‐responsive while some of them can be designed to undergo biodegradation to release drugs follow similar mechanistic pathways as summarized in **Figure** **11**.^[^ ^244^, ^245^ ^]^

### 3.6 Section Summary

Due to their versatile properties, polymeric materials stand unique and most explored for drug delivery nanocarrier design to meet the versatility and complexity of diseases. Among PNCs of various sizes, the ones having sizes less than 200 nm have exhibited the best cellular uptake. Though PNCs of various architectures have been designed, among them the most widely used are nanocomposites, dendrimers, nanogels, nanocapsules, and nanospheres. Polymer nanomicelles are easy to form however their stability is considerably low. Polymersomes can simultaneously encapsulate both the hydrophilic and hydrophobic therapeutics at the core and shell, respectively. However, many times they have shown low encapsulation efficacy and for large scale production it needs a considerably stable set up. Nevertheless, a closer look at these PNCs have revealed that specially the nanomicelles, nanovesicles, nanogels, nanocapsules, etc., have the potential to be explored further for the designing of multi‐drug multi‐stimuli‐responsive delivery systems.

## 4 Physicochemical Properties of Nanocarriers

Besides molecular interactions and environmental stimuli, there are several carrier properties which can also have significant influence on the drug loading and release profiles of PNCs, such as, carrier size, shape and architecture, stability, surface properties, etc. There are various reviews on the roles of environmental stimuli, however evaluations on characters of carriers and their role are underrepresented. Hence, assessments on some of these factors are presented in the following sections to enrich this area.

### 4.1 Dimension

**Figure 12:**
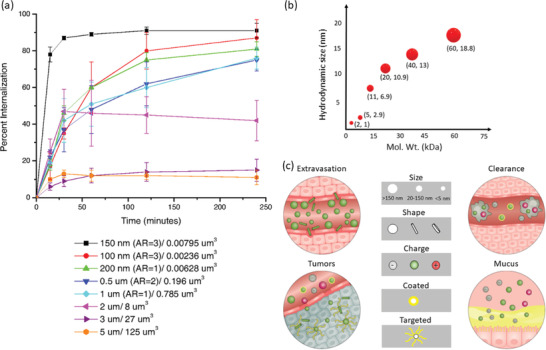
a) Percent internalization of particles having different aspect ratio in HeLa cells. Legends present particle diameter per volume. Reproduced with permission.^[^ ^246^ ^]^ Copyright 2008, The National Academy of sciences of the USA. b) Influence of molecular weight of polyethylene glycol on the size of nanocarriers. Reproduced with permission.^[^ ^249^ ^]^ Copyright 2019, WILEY‐VCH Verlag GmbH & Co. KGaA, Weinheim. c) Fate of nanocarriers depending upon their size, shape, surface charge, and modifications. In general, rod‐shaped nanocarriers extravasate while spherical and larger nanocarriers continue in the circulatory track (top left); nanocarriers having positive surface charge and the ones whose surface are uncoated/modified get cleared out faster by macrophages (top right); neutral, rod‐shaped and targeted nanocarriers can easily permeate tumor showing better local distribution (bottom left) whereas carriers that are smaller, coated, and surface with positive charge can permeate mucosal barriers more easily (bottom right). Reproduced with permission.^[^ ^1^ ^]^ Copyright 2020, Springer Nature.

Dimension of PNCs is an important factor in drug delivery as it influences not only the delivery mechanism but also the drug loading efficacy. With the rapid progress in nanotechnology, carriers of various shapes such as, spheres, stars, rods, cylinders, etc. have been fabricated and applied as nanocarriers for drug delivery. For non‐spherical nanocarriers, aspect ratio (AR) is an important parameter as in many cases it influences the cellular uptake. In a work by Gratton et al., internalization of cylindrical particles having aspect ratio 3 demonstrated about four times faster than that of 1 in HeLa cells despite their volumes were similar and their surface charges were constant (**Figure** **12**a).^[^ ^246^ ^]^ This behavior can be attributed to higher probability of interaction between particle surface and cell membrane facilitated by the large surface area of particles with high aspect ratio. The size of nanomicelles can also influence the drug delivery at targeted sites.^[^ ^247^ ^]^ Cabral et al. have shown that the permeation of polymeric nanomicelle into the tumor cells depends on the sizes of these micelles where they have investigated the accumulations of nanomicelles of sizes 30, 50, 70, and 100 nm on both the poorly, as well as, highly permeable tumors.^[^ ^248^ ^]^ The result showed that nanomicelles of all the sizes permeated the highly permeable pancreatic tumor, however it was only the micelle with 30 nm dimension that could permeate the poorly permeable one. The sizes of polymeric micelles often range from 20 to 200 nm while that of dendrimers is 5–10 nm. Thus, for the cases where smaller size is the primary requirement, one may consider using dendrimer however the loading efficacy might get compromised. The sizes of nanocarriers can be controlled by various factors, such as molecular weight of polymer, extent of intra‐ and inter‐molecular interactions, ionic concentration in the environment, temperature, etc. Recently, Kang et al. have prepared nanocarriers of different sizes (e.g., 1, 2.9, 6.9, 10.9, 13, and 18.8 nm) using PEG of different molecular weights (e.g., 2, 5, 11, 20, 40, and 60 kDa) and investigated their tumor permeation ability (Figure 12b).^[^ ^249^ ^]^ Interestingly, this study found that the nanocarriers having smaller dimensions (<12 nm, ≤20 kDa) achieved significant tumor targeting with almost ignorable nonspecific uptake, whereas the nanocarriers having larger sizes (>13 nm, >20 kDa) accumulated on various major organs including liver, lung, and pancreas. The effective size range of nanocarriers tested by various researchers for treating tumor cells are 10–250 nm and however their efficacy depend on the type of tumor cells, such as, hyperpermeable/hypopermeable, location of the tumor, along with the composition and surface properties of the PNCs.^[^ ^223^ ^]^ Relatively smaller sized nanoparticles favor cellular uptake and subcellular trafficking, however all other factors which are distinct for each unique case, are to be considered to design an effective nanocarrier which is a challenging task.^[^ ^250^ ^]^ Even though larger nanocarriers show relatively better half‐life in circulatory system, it is the smaller ones found to be more efficient in permeating mucosal barrier (Figure 12c).^[^ ^1^ ^]^

### 4.2 Shape and Architecture

Recently, nanocarriers of various shapes and architectures are being progressively investigated to explore their influences on efficacy of drug transport. Majority of the traditionally used PNCs are spherical, nevertheless there is an ever‐increasing interest to investigate a variety of other structures, such as, rod‐, star‐, worm‐like particles, etc.^[^ ^251^ ^]^ Uhl et al. have shown that the long filamentous rod‐shaped nanoparticles exhibited the best therapeutic delivery among the three different shapes (spheres, short rod, and long rod) examined.^[^ ^252^ ^]^ Yang et al. demonstrated dual stimuli‐responsive star‐like hybrid PNCs for hydrophobic drug delivery which showed ≈20.1 wt% drug loading capacity.^[^ ^253^ ^]^ The drug release was stimulated by acidic lysosomal and reducing cytoplasmic environments. Zhang et al. investigated the effects of cylindrical versus spherical shapes on in vitro and in vivo behaviors where they used PNCs made of unimolecular polymers.^[^ ^254^ ^]^ They have found that cylindrical shaped PNCs exhibited improved tissue penetration, higher cellular uptake, and rapid body clearance while the spherical PNCs showed longer circulation time in blood, fast tumor vascular extravasation, and better tumor accumulation. However, the use of elongated or filament‐like morphologies are also being increasingly studied as they can offer extended lifetime in the circulatory system along with higher efficacy of cargo carriage.^[^ ^250^ ^]^ They can reach subcellular target sites quite effectively. Lately, Gao et al. have designed a rod‐like polymeric micelle having its diameter as ≈20 nm and length ≈600 nm which showed half‐life of 24 h in the blood circulatory system and enhanced cellular internalization.^[^ ^255^ ^]^ Star‐like copolymer was used by Wang et al. to design nanocarriers for the delivery of paclitaxel to prostate cancer cells which exhibited biphasic drug release pattern: Initial burst release followed by gradual but continuous release.^[^ ^256^ ^]^

It is known that the architectures of both the nanocarriers and polymers play significant role in determining the success of PNCs.^[^ ^257^, ^258^ ^]^ Tao et al. have prepared two different polymeric micelles, namely dynamic and unimolecular sugar‐based.^[^ ^259^ ^]^ The dynamic micelle was formed by the self‐assembly of amphiphilic polymer molecules while the unimolecular micelle was covalently bound amphiphilic macromolecular micelle. It is observed that, in the unicellular and dynamic micelles the efficacy of loading depended primarily on the hydrodynamic volume of drugs (triclosan and suloctidil). Here, the unimolecular micelle exhibited higher loading capacity in comparison to the dynamic micelle. However, their release behavior did not show strong dependence on the architectures of micelle. When the multi‐molecular amphiphilic polymeric micelles and unimolecular multi‐arm copolymers with dendritic core along with hyperbranched and comb‐like polymers are compared, it is found that the later forms micelles more easily and the stability is much better as they are not susceptible to easy dismantle.^[^ ^260^ ^]^ Though rod‐shaped nanocarriers extravasate faster while the spherical carriers remain longer in the circulatory system, it is the rod shaped nanocarriers that have shown better permeability to tumor cells (Figure 12c). Thus, the variation in the architectures of both the polymer and nanocarriers play critical role in modifying carrier properties.

### 4.3 Stability

A carrier must be sufficiently stable to avoid premature delivery of therapeutics. There are several ways in practice to tune the stability of nanocarriers, for example, optimization of chain length of each block in the copolymer used, effective charge on the polymer chain, type (electrostatic, covalent, H‐bond) and extent of molecular interactions, surface modification of nanocarriers, etc. In one of their current work, Palanikumar et al. prepared a biocompatible and pH responsive hybrid nanocarrier having drug‐loaded core made of PLGA and shell composed of covalently crosslinked bovine serum albumin.^[^ ^261^ ^]^ Presence of this shell diminished contacts of the carrier with serum proteins and macrophages which subsequently increased its lifetime and exhibited improved target recognition. The shell surface was functionalized with acidic pH triggered rational membrane peptide that facilitated specific uptake of the carrier by cancer cells within the acidic tumor microenvironment. Thus, surface modification can not only influence the drug loading efficacy and targeted delivery, but also influence the carrier stability. It is to note that polymer composition, immediate environment around a nanocarrier and drug encapsulation also influence the stability of a carrier.^[^ ^262^, ^263^ ^]^ Thus, to prepare a suitable carrier one must consider all these factors. In 1996, Lemoine et al. had investigated the stability of polymer nanoparticles where it was explored that stability can be influenced by the factors like molecular weight, crystallinity of the polymer, nanocarrier size, and pH.^[^ ^264^ ^]^ The stability of the nanocarriers made of PCL, poly(D,L‐lactide) (PLA), and poly(D,L‐lactide‐*co*‐glycolide) had been investigated. It was observed that the stability of the nanocarriers depended on the type of polymer in the following order: PLA50 = PCL > PLA37.5GA25 > PLA25GA50. The temperatures at which the PNCs were stored also had impact on their stability. It was found that the PNCs made of PLA50 and PCL could be stored at both 4 °C, as well as, room temperature for a year without compromising their stability whereas the PNCs made of PLA25GA50 and PLA37.5GA25 required 4 °C. It was also noticed that the storing the PNCs in buffers or after lyophilizing can improve their stability.

In the cases of stabilities of PNCs less than the required value, the drugs get released prematurely prior to reaching the destination, on the other hand if the stability is too high then the drug would take longer to get released which can be life threatening. Thus, it is very important that the stability of PNC is optimized in each specific case based on the drug and the complexity of the disease.

### 4.4 Surface Properties

Surface properties of a nanocarrier can greatly influence its lifetime in several ways. Once the nanocarrier is administered via intravenous pathway, its interaction with the mucus, epithelia, etc., can determine the lifetime in the circulatory system. Logie, et al. have demonstrated that modifying the hydrophilic corona using PEG, lifetime of the polymeric nanomicelle got improved.^[^ ^265^ ^]^ Subsequently, high density of PEG resisted the dissociation of the micelle in the media having the serum protein. There was almost no dissociation even after 72 h. By changing the number of PEG chains per polymer backbone from 0.5 to 6, the stability was improved. Thus, surface property of nanomicelle can regulate the stability and thereby achieve improved targeted delivery. Surface charge also influences the colloidal stability of nanocarrier‐based drug formulations which thereby impacts the shelf‐life of several medicines. PNCs bearing cationic charges tend to bind to the negatively charged membranes of macrophage prior to the endocytosis. Surface functionalization of PNCs can play crucial role in determining their entry pathways to cell. For example, PNCs having their surface decorated with amphiphilic polymers favors cell penetrating pathway. As per the prerequisites of certain applications, hydrophilic/hydrophobic properties can be introduced to PNCs via surface functionalization. Additionally, the hydrophilicity or hydrophobicity of the surfaces can be regulated by controlling the density of the molecules being used for the functionalization. Using pH‐responsive polymers or small molecules for functionalization, the carriers can inherit the stimuli‐responsive nature. pH is the most explored stimulus in use as factor to trigger drug loading and release from a PNC and excellent reviews are available in various literatures. Recently, simultaneous use of dual‐ or multiple‐stimuli are in the rise. Hiruta et al. have reported dual‐stimuli, that is, temperature‐pH responsive micelle type PNCs used for doxorubicin loading and delivery.^[^ ^266^ ^]^ The micelle is designed having temperature‐responsive surface and endosomal pH‐responsive core. Here, PNC surface which was functionalized by temperature‐responsive polymer facilitated cellular uptake triggered by temperature as external stimulus while the drug release was stimulated by pH, the internal stimulus. Lee et al. designed a polymeric micelle having three factors serving as stimuli—pH, redox, and photo‐responsive properties for the delivery of hydrophobic drug Taxol.^[^ ^267^ ^]^ Here, the surface was prepared as pH and dithiothreitol (DTT)‐responsive. Thus, it is understood that the surface of a nanocarrier can incorporate multi stimuli‐responsive properties which can be foreseen as highly potential for advanced nanobiomedicine design. Introduction of antimicrobial agents to the surface of PNCs can also allow these particles to work as antibacterial agents alongside its drug delivery function. There are various works where improved targeted delivery have been achieved via surface functionalization of PNCs using targeting molecules.^[^ ^268^ ^]^ Generally, nanocarriers having their surfaces uncoated or functionalized by positive charges get easily cleared by microphages whereas targeted and neutral nanocarriers can penetrate tumors more effectively (Figure 12c). It is also to note that nanocarriers having their surfaces positively charged can favorably navigate through mucosal barriers. Thus, surface property of PNCs is a vital factor which can potentially contribute to their colloidal stability, stimuli‐responsive nature, cellular entry, lifetime in circulatory system, and achieving targeted delivery.

### 4.5 Section Summary

PNCs have enormous potential to serve as therapeutic carriers due to the versatility of their component polymers. Nevertheless, the concern about their stability in physiological environment and subsequently premature delivery has always been a tough challenge to address. Thus, to design a suitable PNC, its size, architecture, surface properties, stability, etc., must be considered to achieve efficient drug loading, superior biodistribution, and release at the targeted site. Engineering the surface as well as the core can modify traditionally used PNCs to multiple stimuli‐responsive drug carrier desired for the advanced nanomedicine development. Thus, suitable surface modifications of the PNCs can reduce their interactions with the physiological barriers and improve their efficacy to achieve sustained and targeted delivery. It is to be noted that the PNCs greater than 150 nm tends to be uptaken by phagocytes while the size less than 150 nm can attain longer lifetime in the circulation system. It is also observed that the spherical PNCs can exhibit longer lifetime however the elongated carriers can show better cell penetration. Keeping all these characteristics in view, it is understood that to design an ideal PNC for a particular application, one must precisely control all these parameters so that the carrier can acquire required physical and chemical properties.

## 5 Multi‐Stimuli‐Responsive and Multi‐Drug Delivery: The Way Forward

**Figure 13:**
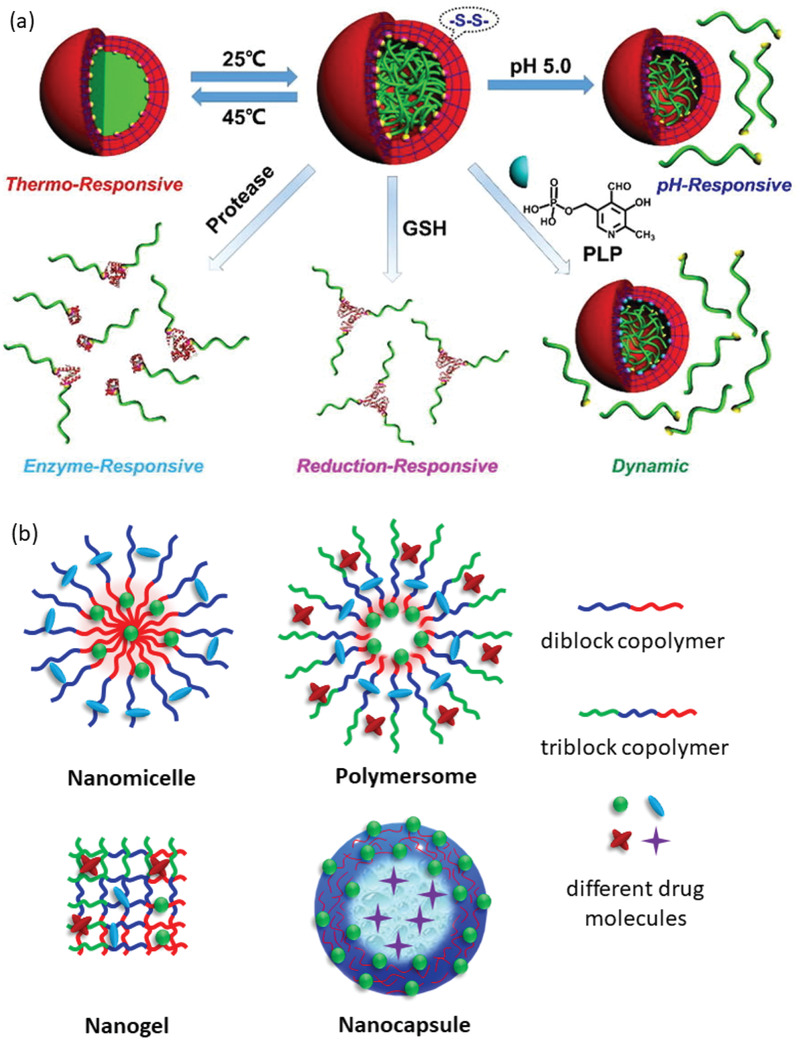
a) A schematic presentation of multi‐stimuli‐responsive biohybrid polymeric nanocarrier. The nanocarrier is responsive to physical, chemical, and biological stimuli. Reproduced with permission.^[^ ^269^ ^]^ Copyright 2017, Elsevier. b) Design strategies of a few representative PNCs for multi‐drug delivery. Various types of drug molecules can be loaded on a carrier based on the properties of polymer blocks.

Development of PNCs having multi‐stimuli‐responsive and multi‐drug delivery properties is the promising future direction. Some of the works have already been carried out toward this direction; however, as of now the progress remained inadequate which is mostly due to the challenges involved in designing these multi‐functional carrier materials, as well as, the limited number of invested research efforts. Wang et al. presented a promising multi‐stimuli‐responsive biohybrid PNC having cross‐linked bovine serum albumin corona which was self‐assembled by copolymer‐protein biodynamer (**Figure** **13**a).^[^ ^269^ ^]^ The pyridylhydrazone linkers used in this work was cleaved in an acidic milieu which was a mimic of the intracellular environment and subsequently the protein was released. Here, the protein biodynamer self‐assembled into micelle type PNC above its lower critical solution temperature (LCST). At temperature 45 °C, the protein molecules were crosslinked via cystamine inside the hydrophilic corona. This biohybrid PNC exhibited reversible swelling and shrinking as the core got solvated below LCST while above this temperature it was desolvated. Additionally, the reversibility of pyridydrazone bonds imparted pH‐responsive nature to the PNC. This PNC also exhibited glutathione‐responsive nature. The synergistic nature of glutathione and pH resulted in the complete disintegration of the carrier under the intracellular‐mimicking reductive and acidic condition. Thus, this corona cross‐linked PNC has all the physical‐, chemical‐, and biological‐ stimuli‐responsive properties which shows the direction to which the future nanocarrier design should move to develop advanced nanomedicine. Recently, Jiang et al. have also designed a multi‐stimuli‐responsive nanomicelle composed of pH, photo (UV and visible), and temperature responsive poly[(2‐dimethylaminoethyl) methacrylate]‐*co*‐poly(spiropyan‐functionalized) material.^[^ ^270^ ^]^ Similarly, a triple‐stimuli‐responsive (pH, temperature, and glucose) poly[*N*‐2(3‐pentadecylphenoxy)ethylmethacrylamide]‐*co*‐poly[2‐(2‐(2‐methoxyethoxy)ethoxy)ethyl methacrylate]‐based hydrogel was prepared by Wang et al. and was applied for the controlled release of bovine serum albumin.^[^ ^271^ ^]^ It is to note that designing a single polymer chain having different stimuli‐responsive properties is often a challenging task. Additionally, introduction of multi‐drug release property into the same polymeric molecule makes the development further difficult. At this juncture, role of copolymer is pivotal as they can be composed of several polymeric blocks responsive to various stimuli and having drug loading/release properties (Figure 13b). Following the demands of a carrier, several such polymeric blocks can be chosen judiciously and then copolymerized to form a polymer molecule smart enough to be multi‐functional. Thus, these polymeric materials are highly potential to design sophisticated multi‐stimuli‐responsive multi‐drug delivery advanced nanocarriers.

The advantages of polymers to design such multi‐functional nanocarriers are also the availability of polymers that are responsive to pH, temperature, light, glucose, redox potential, enzyme, etc. Various examples of such polymers belonging to different categories, such as, polyether, poly(carboxylic acid), poly(amino acid), poly(sulfonic acid), poly(phosphoric acid), poly(boronic acid), natural polymer, polymers containing 3°‐amine, imidazole, pyridine, pyrrolidine, piperazine groups, and dendrimer are available in various literatures. A list of some polymers along with their stimuli‐responsive natures and the types of carriers prepared using them are presented in **Table** **1**. The list has a few representative polymer candidates having huge potential for further exploration to design multi‐stimuli‐responsive multi‐drug delivery PNCs.

**Table 1:** A representative list of polymers which can further be explored for multi‐stimuli‐responsive and multi‐drug delivery polymeric nanocarrier design; (a)Full forms of the abbreviated words are provided at the bottom of the table)

Molecular structures	Names	Stimulia)	Types of carriersa)	Ref.
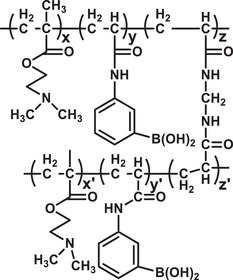	Poly[(2‐dimethylaminoethyl) methacrylate]‐*co*‐Poly(3‐acrylamidophenylb‐oronic acid)	Glucose, Temp., pH	Gel	^[^ ^271^ ^]^
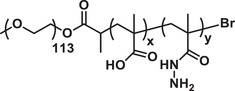	Polyethylene oxide‐*b*‐poly(methacrylic acid)‐*g*‐hydrazine	pH, Red. Pot	Micelle	^[^ ^272^ ^]^
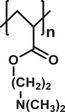	Poly(2‐(Dimethylamino)et‐hyl methacrylate)	Temp., pH	NP	^[^ ^273^ ^]^
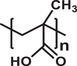	Poly(acrylic acid)	pH	NP	^[^ ^274^ ^]^
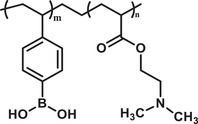	Poly(vinylphenyl boronic acid)‐*co*‐poly[(2‐dimethylaminoethyl) ethacrylate]	Glucose, pH	Nanogel	^[^ ^275^ ^]^
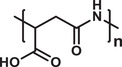	Poly(aspartic acid)	pH	NP	^[^ ^276^ ^]^
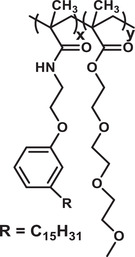	Poly[N‐2(3‐pentadecylphenoxy)ethyl methacrylamide]‐*co*‐poly[2‐(2‐(2‐methoxyethoxy)et‐hoxy)ethyl methacrylate]	Temp., Enzyme	Micelle	^[^ ^277^ ^]^
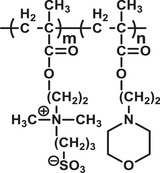	Poly[sulfobetaine(2‐dimethylaminoethyl) methacrylate]‐*b*‐Poly(2‐N‐morpholinoethyl)m‐ethacrylate	pH, Temp., Salt	Micelle	^[^ ^278^ ^]^
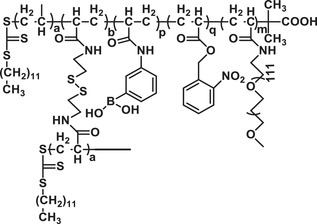	Poly(methoxypolyethylene glycol acrylamide)‐*b*‐poly(2‐nitrobenzyl acrylate‐*co*‐poly(3‐acrylamidophenylb‐oronic acid)	Glucose, Photon	Micelle	^[^ ^279^ ^]^
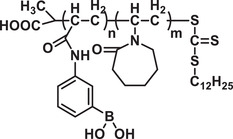	Poly(3‐acrylamidophenylb‐oronic acid‐block‐*N*‐vinyl caprolactam)	Glucose	NP	^[^ ^280^ ^]^
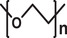	Poly(ethylene glycol)	Photon	Gel	^[^ ^281^ ^]^
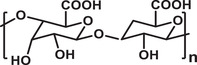	Alginic acid	pH	NP	^[^ ^282^ ^]^
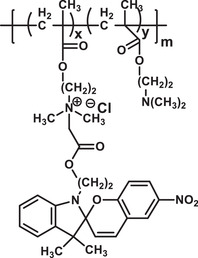	Poly[(2‐dimethylaminoethyl) methacrylate]‐*co*‐Poly(spiropyan‐functionalized)	Photon, Temp., pH	Micelle	^[^ ^270^ ^]^
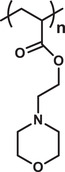	Poly[2‐(*N*‐morpholino) ethyl methacrylate]	pH, Temp., Salt	Gel	^[^ ^283^ ^]^
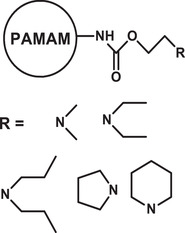	Poly(amidoamine) dendrimers with peripheral *N*‐dialkylaminoethyl carbamate moieties	Temp., pH, Salt	NP	^[^ ^284^ ^]^

Temp.: Temperature; NP: Nanoparticle; Red.: Redox potential; PAMAM: Poly(amidoamine) dendrimer.

**Figure 14:**
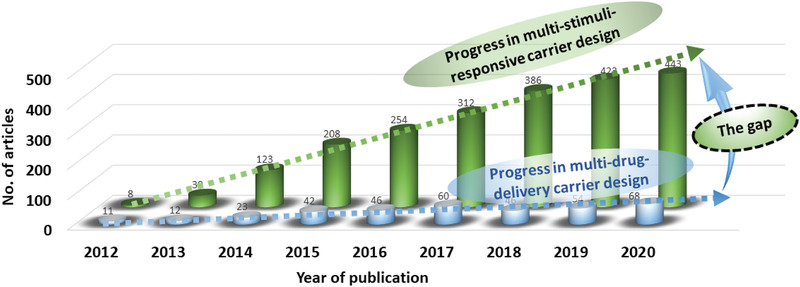
An overview on the work progress in multi‐stimuli‐responsive and multi‐drug‐delivery polymeric nanocarrier design. The literature searches were carried out in Scopus database. Keywords used for the searches are “polymer” + “multi‐stimuli‐responsive” and “polymer” + “multi‐drug‐delivery.”

At present, research interests on designing multi‐stimuli‐responsive PNCs are steadily rising while efforts for multi‐drug‐delivery system design are yet to pick up the pace, as per the information available from the relevant publications (**Figure** **14**). Thus, so far there are not many carriers designed which has both the multi‐stimuli‐responsive and multi‐drug loading/release properties incorporated into a single carrier, however the demand of such PNCs in the clinical application field is sufficiently high. Thus, with the progress on producing multi‐functional polymers along with the recent advances in nanotechnology, designing of such multi‐stimuli‐responsive multi‐drug‐delivery PNCs is anticipated to revolutionize the field of nanomedicine.

## 6 Conclusions and Perspectives

This review has presented a succinct analysis on structure‐based variety of PNCs optimized for the loading and delivery of therapeutics of diverse properties, and the carriers are responsive to pH, ions, sonication, temperature, redox potential, glucose, enzyme, photon, etc. It is also found that some of the recent works are engaged to incorporate multi‐stimuli‐responsive, as well as, multi‐drug delivery properties into the PNCs. Availability of numerous natural sources, ease of synthesis, and versatile physicochemical properties of polymer have made these macromolecules one of the most appealing materials to design customized therapeutic carriers so that the challenges posed by complexity of disease, patient heterogeneity, and comorbidity can be overcome. Additionally, substantial progress in nanotechnology associated with engineering drug carriers are providing several innovative prospects which has the potential to lead further exploration to design PNCs to deal with the challenges posed by biological barriers and traffic therapeutic cargoes. PNCs offer huge platform to design carriers of different sizes, shapes, architectures, surface properties, and stimuli‐responsive nature. Suitable choice of polymers from the vast library or synthesizing them widens the opportunities to design the nanocarriers with desired properties. Recently, various research groups have focused on designing PNCs which can have the multi‐drug loading and release capability in a controlled manner. Dual drug delivery systems that can simultaneously load and carry genetic therapy substances and cancer drug have already been developed. Moreover, recent developments of strategies that can prepare nanocarriers as multi stimuli‐responsive have added significant value to the growing field of nanomedicine. Thus, the future target would be to combine these properties and design intelligent nanocarriers which would be multifunctional. Several works have demonstrated that PNCs can also be designed to serve both the imaging and drug delivery purposes, simultaneously. It is also established that PNCs can protect ultra‐sensitive cargos, such as, therapeutic proteins, DNA, etc. against harsh environment (e.g., elevated temperature) or enzymes.^[^ ^285^ ^]^ Hence, PNCs are potential candidates for vaccine delivery to combat viral diseases like COVID‐19, etc. It is identified that the PNCs having size ≤ 100 nm, negatively charged or neutral surfaces can demonstrate enhanced half‐life in circulatory system. In many of the cases, nanoparticles having higher aspect ratio obtained better internalization by cell in comparison to the spherical ones. Here, it is to note that kidneys can easily clear out nanoparticles of size less than 10 nm through excretion, hence, to improve the circulation half‐life PNCs of size greater than 10 nm would be effective and, in such cases, biodegradable PNCs are the most preferred nanomaterials.

Nevertheless, since the design procedures of multi‐functional nanocarriers are often complicated, hence before generalizing the claims and their trends in drug delivery applications, more investigations across large populations and their heterogeneity would be required. Furthermore, emphasis must be given on designing biodegradable carriers as they will not have any adverse impact on normal cells, tissues, or organs and at the same time there will be no requirement of any other arrangement to clear those from the body. However, at present more data are to be collected through further research to develop better understanding on designing multipurpose PNCs to diagnose and treat diseases, here collaborating with artificial intelligence (AI) can also contribute to accelerate the progress. At present, majority of the studies investigate therapeutic loading and release behavior based on the interactions of PNCs with their physiologically relevant environmental parameters, however, influence of the physicochemical properties (e.g., size, stability, architecture, and surface properties) of PNCs also need to be considered during the development of multi‐stimuli‐responsive multi‐drug delivery nanocarriers. Another very crucial reason for the limited success of PNCs in clinical application is that majority of the carriers are screened for their efficacy of drug loading and targeted delivery in broad populations where getting heterogeneity of patients remained a big challenge.

It is anticipated that with the continuous advancements in nanocarrier design along with growing interests to develop multi‐stimuli‐responsive and multi‐drug delivery PNCs, opportunities will multiply to integrate numerous promising drug candidates into the clinical trials. These PNCs should also have the ability to permeate through biological barriers, whenever required. Ever increasing diversity of diseases along with their complex natures are advocating developments of nanocarriers which would provide a platform to achieve multiple purposes and subsequently minimize the need of personalized delivery carriers, that is, single carrier will be able to execute multiple functions. Thus, this review establishes that designing intelligent PNCs having multi‐stimuli‐responsive multi‐drug delivery capability is the future direction.

However, it is to note that despite of growing research interests and availability of currently developed different multifunctional PNCs, their complete clinical transition have not yet been achieved as there are various critical challenges to overcome. For example, the sensitivity (response time) and stability of these PNCs in vivo are to be improved. Though various successes have been achieved in vitro, their utilization in vivo is more complicated, and hence further investigations are required. Furthermore, the polymeric materials are to be biocompatible and biodegradable, and the desired molecular weights of such macromolecules is below 40 kDa to achieve good renal clearance to avoid immunogenicity.^[^ ^286^, ^287^ ^]^ However, natural availability of such polymeric materials are limited, hence they are to be synthesized and their physicochemical properties are to be tuned by adjusting their molecular composition, conformation, charge, flexibility, etc.^[^ ^288^, ^289^ ^]^ In addition, the polymers are also to be rationally designed to have the ability to permeate through biological barriers, such as, skin, BBB, mucosa, etc. Thus, to achieve full translational prospects, these parameters should be considered when multifunctional PNCs are designed for in vivo investigations. Again, it is to note that overcomplicated steps involved in designing such multifunctional PNCs will make it impractical to translate the achievements into clinical practices. In fact, the complicated and multiple steps involved in designing a multi‐stimuli responsive multi‐drug delivery PNC are the hardest challenges to overcome for their large‐scale production. Though, some of the external stimuli, such as, light, sound, etc., can be regulated manually and the impacts are comparatively reproducible, the endogenous stimuli often generate inconsistent outcomes. Here, the nanomaterials are to be engineered in such a way that they can be sufficiently sensitive and result precision‐controlled therapeutic delivery. A better understanding about the influence of physiological stimuli on mechanistic pathways of drug delivery is also required when the multifunctional PNCs are designed. However, further collaborative efforts of the scientists from the fields of nanotechnology, chemistry, biology, and medicine are required to improve the efficiency of multi‐stimuli responsive multi‐drug delivery PNCs and to translate their potential into complete clinical realization which can revolutionize the field of drug delivery.

## Conflict of Interest

The authors declare no conflict of interest.

## Author Contributions

R.D. conceived and drafted the manuscript, prepared figures, and summarized the table. R.D. and M.K.M. discussed the concepts and edited the final version. K.‐T.K. supervised, edited, and approved the version for submission.
